# Global extent of chloroquine-resistant *Plasmodium vivax*: a systematic review and meta-analysis

**DOI:** 10.1016/S1473-3099(14)70855-2

**Published:** 2014-09-08

**Authors:** Ric N Price, Lorenz von Seidlein, Neena Valecha, Francois Nosten, J Kevin Baird, Nicholas J White

**Affiliations:** a Worldwide Anti-malarial Resistance Network, Nuffield Department of Clinical Medicine, University of Oxford, Oxford, UK; b Centre for Tropical Medicine, Nuffield Department of Clinical Medicine, University of Oxford, Oxford, UK; c Global Health Division, Menzies School of Health Research, Charles Darwin University, Darwin, NT, Australia; d National Institute of Malaria Research, Dwarka, New Delhi, India; e Shoklo Malaria Research Unit, Mae Sot, Tak, Thailand; f Mahidol-Oxford Tropical Medicine Research Unit, Bangkok, Thailand; g Eijkman-Oxford Clinical Research Unit, Jakarta, Indonesia

## Abstract

**Background:**

Chloroquine is the first-line treatment for *Plasmodium vivax* malaria in most endemic countries, but resistance is increasing. Monitoring of antimalarial efficacy is essential, but in *P vivax* infections the assessment of treatment efficacy is confounded by relapse from the dormant liver stages. We systematically reviewed *P vivax* malaria treatment efficacy studies to establish the global extent of chloroquine resistance.

**Methods:**

We searched Medline, Web of Science, Embase, and the Cochrane Database of Systematic Reviews to identify studies published in English between Jan 1, 1960, and April 30, 2014, which investigated antimalarial treatment efficacy in *P vivax* malaria. We excluded studies that did not include supervised schizonticidal treatment without primaquine. We determined rates of chloroquine resistance according to *P vivax* malaria recurrence rates by day 28 whole-blood chloroquine concentrations at the time of recurrence and study enrolment criteria.

**Findings:**

We identified 129 eligible clinical trials involving 21 694 patients at 179 study sites and 26 case reports describing 54 patients. Chloroquine resistance was present in 58 (53%) of 113 assessable study sites, spread across most countries that are endemic for *P vivax*. Clearance of parasitaemia assessed by microscopy in 95% of patients by day 2, or all patients by day 3, was 100% predictive of chloroquine sensitivity.

**Interpretation:**

Heterogeneity of study design and analysis has confounded global surveillance of chloroquine-resistant *P vivax*, which is now present across most countries endemic for *P vivax*. Improved methods for monitoring of drug resistance are needed to inform antimalarial policy in these regions.

**Funding:**

Wellcome Trust (UK).

## Introduction

*Plasmodium vivax* is a major cause of morbidity, causing 72–390 million clinical cases of malaria worldwide each year.^1^, ^2^ Vivax malaria is an important cause of morbidity, especially in young children, with adverse consequences for education, development, and wellbeing. Unlike *Plasmodium falciparum* malaria, *P vivax* forms dormant liver stages (hypnozoites), which cause relapses of infection weeks to months after the initial attack. In some areas, recurrent infections can occur as often as every 3 weeks, with relapses the main cause of vivax illness. Acute febrile episodes are associated with anaemia, intrauterine growth retardation,^3^ miscarriage, and severe and fatal disease.^4^, ^5^, ^6^, ^7^

Policy makers and malaria researchers have generally focused on *P falciparum*, which is the main cause of malaria mortality. Substantial progress has been made in reduction of the global burden of malaria, much of which has been attributed to increases in access to health-care services, early diagnosis, treatment with highly effective antimalarial drug regimens, and deployment of insecticide-treated bednets. One of the greatest threats to control and elimination efforts is the emergence and spread of antimalarial drug resistance. This recurring problem has plagued malaria therapeutics for more than 60 years. In the early 1950s, chloroquine became established as the best antimalarial drug for all human malarias, but within a decade resistance had emerged. Chloroquine-resistant *P falciparum* spread throughout malaria-endemic countries killing millions of people. Antimalarial drug resistance is measured by the prevalence and severity of treatment failure. In *P falciparum* malaria recrudescent infection is defined as the re-emergence of a genetically identical parasite in the peripheral blood after its initial treatment with a usually curative drug regimen (thereby distinguishing it from a newly acquired infection). *P vivax* clinical efficacy studies are more difficult to interpret, because recurrent infections can arise from recrudescence, reinfection, or relapses (arising from the dormant liver stages).^8^ Relapses can be prevented only by 8-aminoquinolines such as primaquine.

Chloroquine is the first-line treatment for *P vivax* malaria in most endemic countries. When given with primaquine (radical cure), the combination is highly effective against both the acute illness and in prevention of relapses from hypnozoites. Chloroquine-resistant *P vivax* was first reported in 1989, almost 30 years after chloroquine-resistant *P falciparum* was first noted.^9^, ^10^ The absence of reliable, robust, sensitive methods for detection, mapping, and monitoring of antimalarial drug efficacy in *P vivax* has almost certainly contributed to the delayed recognition of this emerging problem.^11^ This delay has had important public health implications. In areas where high-grade chloroquine-resistant *P vivax* is prevalent (such as Indonesia and Oceania), partly effective drug treatments and consequent recurrent infections are an important contributing factor to severe anaemia from *P vivax* malaria.^12^ Recognition and definition of the scale of the problem is key for treatment guidelines to be revised and appropriate control and elimination strategies to be devised and implemented. In this Article, we present a systematic review and meta-analysis of *P vivax* drug trials that summarises the geographical extent and level of evidence for reduced *P vivax* susceptibility to chloroquine and estimate risk of recurrence by day 28.

## Methods

### Study design

Our analysis adhered to the recommendations of the Preferred Reporting Items for Systematic Reviews and Meta-Analyses (PRISMA) guidelines.^13^ We systematically searched Medline, Web of Science, Embase, and the Cochrane Database of Systematic Reviews to identify studies of chloroquine treatment of vivax malaria published in English between Jan 1, 1960, and April 30, 2014. A complete list of the search terms and the antimalarial clinical trials identified is presented online by the WorldWide Antimalarial Resistance Network (WWARN).^14^ We filtered this database for clinical studies containing “vivax” in the title or abstract. Reference lists of previously published reviews and papers on chloroquine-resistant *P vivax* were also screened for relevant studies in English.

We systematically extracted data on study characteristics, recurrence, and side-effects from the articles and entered into an EpiInfo database (version 3.5.1). We extracted the study method, including location of study site, inclusion and exclusion criteria, clinical setting, randomisation and masking, and the chloroquine dose regimen (dose, frequency, duration, and supervision of treatment), and the co-administration of primaquine; these data are available online.^15^ We identified study sites with Google Earth. We excluded antirelapse studies that did not include a treatment group without primaquine, and studies in which treatment was not supervised.

We extracted estimates of chloroquine efficacy and present them separately for each treatment group at each study location (termed site estimates). The primary outcome measure was the risk of recurrent *P vivax* parasitaemia at day 28. Results of previous studies have shown that the prolonged elimination of chloroquine provides blood concentrations that prevent recurrence of chloroquine-sensitive *P vivax* for about 35 days. Hence, no recurrent parasitaemia should be noted within 28 days of treatment in patients taking a complete treatment course with adequate absorption.^16^ The proportion of patients with recurrent *P vivax* parasitaemia by day 28 is therefore a useful measure of chloroquine resistance, whether recurrence is caused by recrudescence, relapse, or a new infection. Secondary outcome measures included the proportion of patients with parasitaemia on days 1, 2, and 3; the day of the first recurrence; and the proportion of patients with early treatment failure, defined according to WHO criteria for *P falciparum.*^8^, ^17^

We separated chloroquine resistance a priori into four categories according to the primary outcome. Category 1 resistance constituted greater than 10% recurrences by day 28 (with a lower 95% CI of >5%), irrespective of confirmation of adequate blood chloroquine concentration; occasional breakthrough recurrences do occur within 28 days of chloroquine treatment, but a risk greater than 10% in a large enough sample is very suggestive of resistance. Category 2 resistance included confirmation of recurrences within 28 days, in the presence of whole-blood chloroquine concentrations greater than 100 nm;^16^ parasite growth in the presence of high blood concentrations of the drug confirms resistance. Category 3 resistance included at least 5% recurrences by day 28 (lower 95% CI of <5%), irrespective of confirmation of adequate blood chloroquine concentration; this category suggests potential evidence of chloroquine resistance, but might result from other factors such as poor drug absorption or drug quality. In category 4, chloroquine sensitivity was confirmed if patients had enrolled after a symptomatic clinical illness, fewer than 5% recurrences had occurred by day 28, no primaquine was given before day 28, and studies had a sample size of at least ten patients.

### Statistical analysis

We restricted analysis of drug efficacy to patients treated with a chloroquine regimen. We calculated the risk of recurrence per protocol with the number of patients with recurrent *P vivax* during 28 days of follow-up, with the denominator being the number of patients followed until the study endpoint. We estimated the risk of recurrence for each chloroquine group, stratified by study location. We estimated the effect of primaquine by comparing recurrence rates in randomised studies in which patients were treated with and without concomitant primaquine. We estimated the comparative risk of recurrence by day 28 as Mantel-Haenszel odds ratios with Review Manager version 5.2. We analysed all data with SPSS v19.0 for Windows.

### Role of the funding source

The funder had no role in study design, data collection, data analysis, data interpretation, or writing of the report. RNP had full access to all the data in the study, and had final responsibility for the decision to submit for publication.

## Results

**Figure 1:**
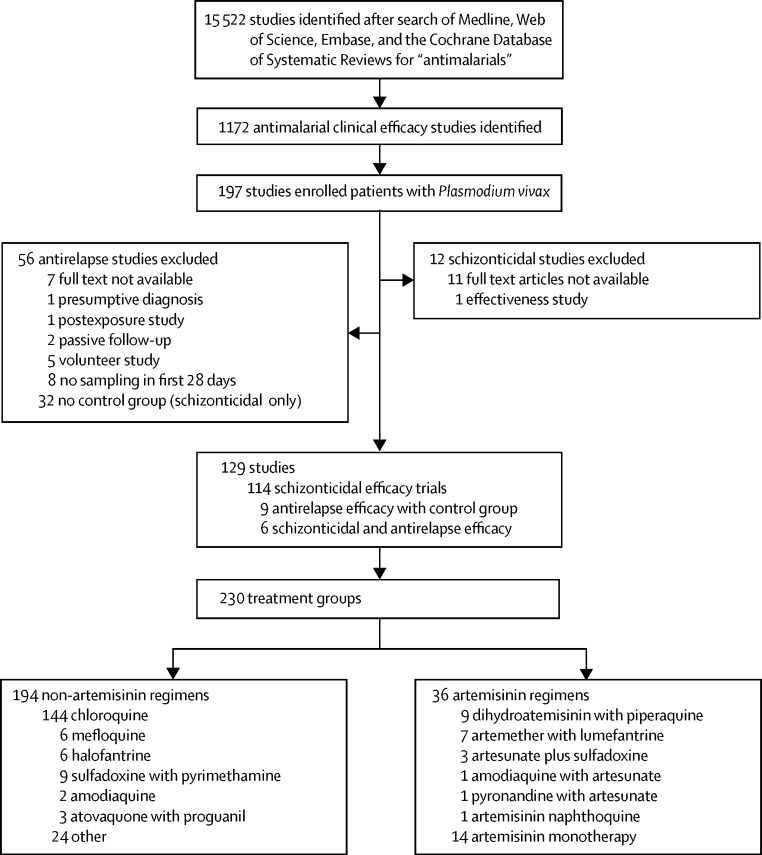
Study selection

We identified 198 clinical trials enrolling patients with *P vivax*, of which we excluded 68 (figure 1). Of the 129 clinical trials included, 115 were designed primarily to establish efficacy against *P vivax* blood stages (schizonticidal activity). 14 studies were designed as antirelapse studies to assess the radical curative efficacy of primaquine or tafenoquine, but also enrolled a chloroquine-only comparative treatment group (nine studies) or were designed to estimate both schizonticidal and antirelapse efficacy (six studies). In 89 (69%) of 129 studies, patients were recruited from outpatient clinics; however, in 19 (15%) studies, patients were enrolled from hospitals, and in 21 (16%) studies, patients were recruited from cross-sectional surveys. Patients recruited from surveys had lower baseline parasitaemia (median 799 per μL, IQR 367–1043) than did those recruited from hospital (9830 per μL, IQR 5634–13 830; p=0·002), or outpatient clinics (4057 per μL, IQR 2800–5514; p=0·002). We assessed 230 treatment groups at 179 study sites, enrolling 21 694 patients. Chloroquine was included in 144 treatment groups (63%; 15 455 patients), sulfadoxine with pyrimethamine in nine treatment groups (3·9%; 486 patients), dihydroartemisinin with piperaquine in nine treatment groups (3·9%; 1132 patients), artemether with lumefantrine in seven treatment groups (3·0%; 1092 patients), mefloquine in six treatment groups (2·6%; 377 patients), and halofantrine in six treatment groups (2·6%; 222 patients).

We noted heterogeneity in the design of the clinical studies (table 1). 55 publications reported comparative drug trials, of which 45 (82%) were randomised. The median number of comparative treatment groups was two (range 2–10). Age-related inclusion criteria were recorded for 87 (67%) studies; children younger than 5 years were excluded from 48 (37%) studies. Parasite density was an enrolment criterion in 52 (40%) studies, the inclusion threshold varying from at least 40 asexual parasites per μL to at least 5000 per μL. Patients with recent treatment were excluded from 52 (40%) studies. The median duration of follow-up was 28 days (range 14–365). Venous drug concentrations were measured in 39 (30%) studies, and genotyping was applied to the analysis of ten (8%) studies.

**Table 1:** Characteristics of selected studies

	**Clinical studies**	**Treatment groups**	**Site estimates of chloroquine efficacy**
**Location**			
Asia	104	195	134
Africa	10	13	13
South America	14	16	22
Multicentre	1	6	8
Overall	129	230	177
**Year of publication**			
1981–90	6	19	8
1991–00	34	68	41
2001–10	65	105	80
2011–14	24	38	48
**Origin of patients enrolled**			
Community surveys	21	25	22
In hospital	19	46	21
Outpatient setting	89	159	134
Follow-up period*			
<27 days	4	4	3
27–34 days	98	163	128
40–45 days	10	21	13
56–63 days	3	6	9
>80 days	12	34	23

* Maximum duration of follow-up not stated in two studies.

The standard dose was a 3 day regimen of chloroquine (total dose 25 mg base/kg), although in eight treatment groups (583 patients) the same total dose was spread over four doses, and in five treatment groups (1595 patients) only a single dose of 7·5–10 mg/kg of chloroquine was given. In four treatment groups, chloroquine was given with sulfadoxine and pyrimethamine, and in one group given with a 7 day course of doxycycline. Primaquine was given in 73 (51%) chloroquine treatment groups: a very high dose (13·5 mg/kg total) in two groups, a high dose (6–7 mg/kg total dose) in eight groups, a low dose (2·5–4 mg/kg total dose) in 49 groups, and a very low dose (1·3 mg/kg total dose) in nine groups.^14^ The primaquine dose was not stated in five publications. The course of primaquine was started at the same time as chloroquine treatment in 21 groups, at the end of chloroquine treatment in 24 groups, and at the end of the study in 23 groups. In five groups primaquine was started during follow-up on day 7 (one group), day 14 (three groups), and day 28 (one group).

**Figure 2:**
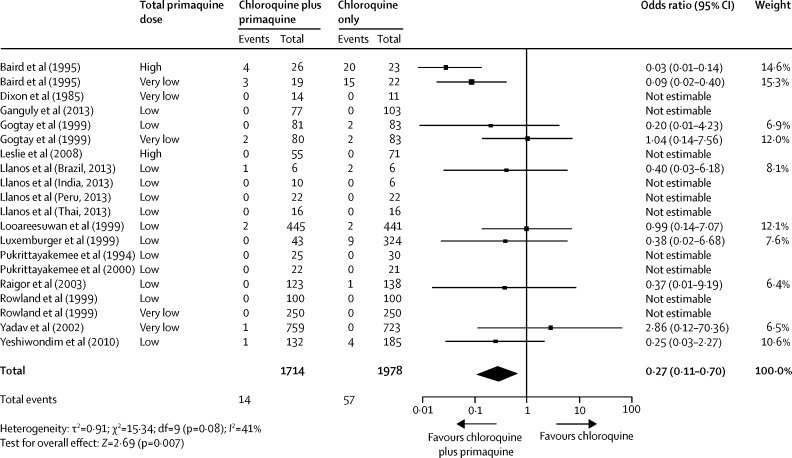
Forest plot of the risk of recurrence at day 28 in patients treated with chloroquine or chloroquine plus primaquine High dose >6 mg/kg. Low dose 3–5 mg/kg. Very low dose <2 mg/kg. As part of a sensitivity analysis, studies in which no recurrences were recorded before day 28 were assigned a numerator of 1, the derived odds ratio was recalculated as 0·43 (95% CI 0·24–0·79; p=0·006). References for all studies are shown in the appendix.

14 studies included patients recruited into comparative studies of chloroquine monotherapy and chloroquine plus primaquine administered at the start of treatment. In three of these studies, two comparisons were made, and in two studies, four comparisons were made. In all but two of these studies patients receiving chloroquine plus primaquine had an equivalent or lower risk of *P vivax* recurrence by day 28 than did patients receiving chloroquine alone (figure 2).

Estimates of chloroquine efficacy could be derived for 177 sites from 144 treatment groups. In 65 (37%) of these sites chloroquine resistance could not be categorised for the following reasons: early administration of primaquine (37 sites); no documentation of parasitological data at day 28 (15 sites); sample size of fewer than ten patients (four sites); good response documented in asymptomatic patients (four sites); and administration of only a single dose of chloroquine (five sites). Overall *P vivax* was defined as chloroquine resistant for 57 (51%) of 112 site estimates, 35 (61%) of these sites fulfilled the predefined criteria for category 1 resistance, eight (14%) for category 2 resistance, and 14 (25%) for category 3 resistance. The median risk of recurrence in these chloroquine-resistant sites was 16·7% (IQR 9·8–31·4). The earliest treatment failure occurred at a median of 14 days (range 3–28), and the interval was negatively correlated with both delayed parasite clearance (as defined by the proportion of patients with peripheral parasitaemia at 48 h; *r*_s_=–0·72, p=0·002), and the risk of recurrent infections at day 28 (*r*_s_=–0·58, p=0·005). These correlations remained significant after controlling for the place from which patients were recruited (hospital, outpatient, or survey).

**Figure 3:**
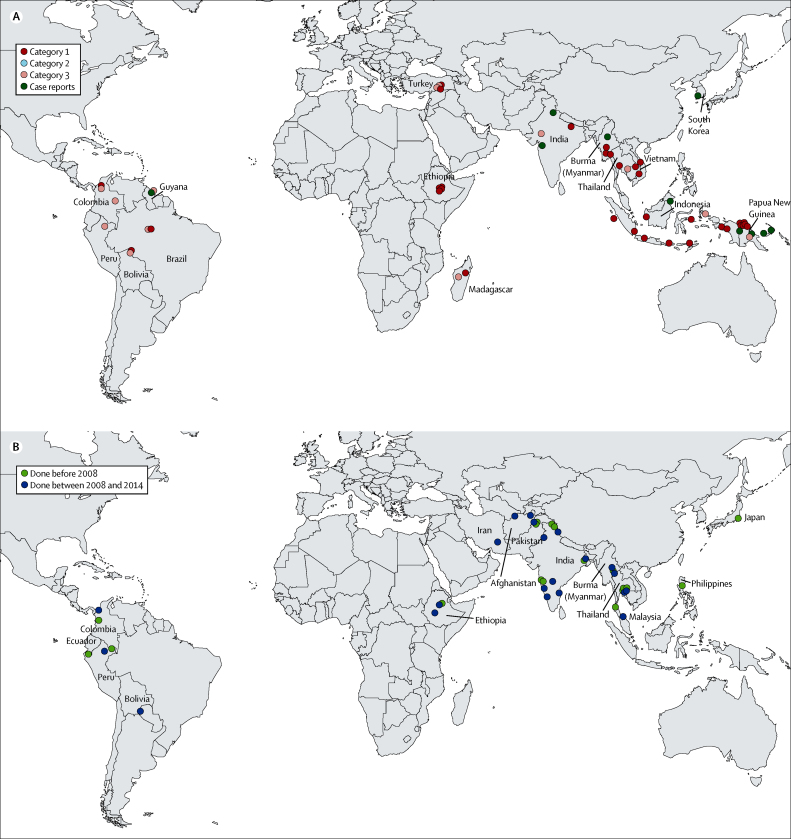
Location of study sites with documented chloroquine-resistant (A) and chloroquine-sensitive *Plasmodium vivax* (B)

**Figure 4:**
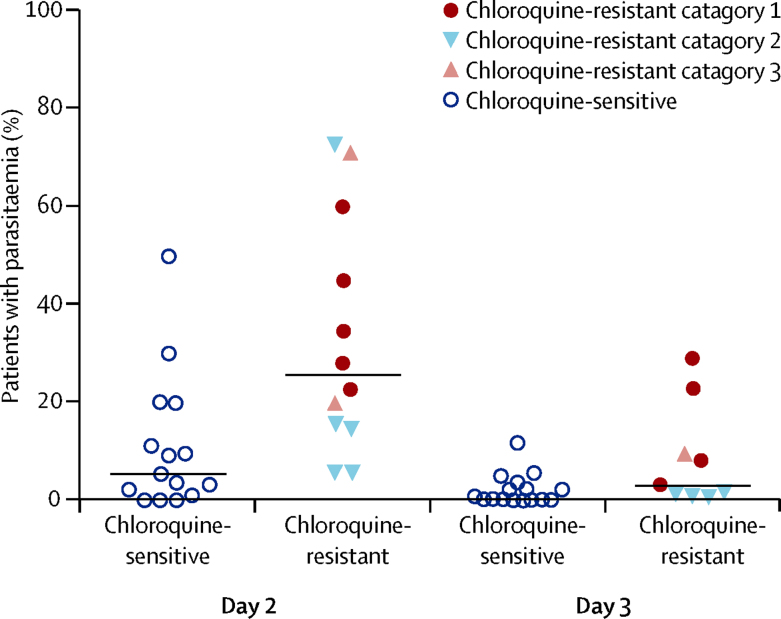
Proportion of patients with parasitaemia on days 2 and 3

Measures of early parasite clearance were documented in 99 (56%) of 177 site estimates, although the actual proportions of patients who had parasitaemia on days 1, 2, and 3 could be deduced for only 60 (61%) of these site estimates. Sites where *P vivax* was categorised as being chloroquine-resistant (figure 3) had a significantly higher proportion of patients with parasitaemia on day 2 (median 25·5%, range 6·0–73·0) compared with those categorised as chloroquine-sensitive (median 7·0%, range 0–50·0; p=0·006; figure 4). The difference was also evident at day 3 (3·0%, 0·9–29·0) for chloroquine-resistant sites *vs* 0·4%, 0–13·7 for chloroquine-sensitive sites; p=0·023). Clearance of parasitaemia (assessed by microscopy) in all patients by day 3, or in 95% of patients by day 2, was 100% predictive of chloroquine sensitivity in the study population as defined by day 28 recurrence.

We identified 26 case reports of 54 patients with suspected chloroquine-resistant *P vivax* (appendix). In 18 patients had breakthroughs during chloroquine prophylaxis were described (five in the presence of high blood chloroquine concentrations), twelve patients had reports of prolonged parasite clearance (eight in the presence of high blood chloroquine concentrations), and 24 patients had recurrence of *P vivax* after treatment (seven with blood-chloroquine concentrations exceeding 100 ng/mL at the time of recurrence). 20 cases had confirmed parasite growth in blood chloroquine concentrations exceeding 100 ng/mL (figure 3). Table 2 shows the publications referring to clinical trials and case reports with patients having early treatment failure after chloroquine.

**Table 2:** Reports of early parasitological failures after chloroquine treatment of *Plasmodium vivax*

	**Type of Report**	**Country**	**Location**	**Number of patients**	**Early treatment failure (%)**	**Day 28 recurrence (%)**	**Comments**
Osorio et al (2007)	Trial	Colombia	Tarapaca	22	5·0%	10·0%	..
Ketema et al (2011)	Trial	Ethiopia	Halaba	87	4·6%	13·8%	..
Tulu et al (1996)	Trial	Ethiopia	Debre Zeit	459	2·0%	2·0%	..
Dunne et al (2005)	Trial	India	New Delhi	102	2·0%	1·0%	Early primaquine
Saravu et al (2012)	Trial	India	Manipal	110	1·0%	1·1%	Early primaquine
Singh et al (2000)	Trial	India	Daltonganj	75	6·7%	22·7%	Early primaquine
Srivastava et al (2008)	Trial	India	Pansora	75	9·2%	9·2%	..
Baird et al (1997)	Trial	Indonesia	Nabire	23	5·9%	70·4%	..
Maguire et al (2006)	Trial	Indonesia	Armopa, Papua	232	20·0%	18·4%	Early primaquine
Ratcliff et al (2007)	Trial	Indonesia	Timika	40	16·0%	72·7%	..
Sumawinata et al (2003)	Trial	Indonesia	Arso	29	24·0%	100·0%	..
Sutanto et al (2009)	Trial	Indonesia	Alor	36	13·8%	43·3%	..
Sutanto et al (2010)	Trial	Indonesia	Lampung	31	9·7%	65·2%	..
Tjitra et al (2002)	Trial	Indonesia	Genyam	9	22·2%	88·9%	..
Than et al (1995)	Trial	Burma	Mingaladon	50	6·0%	14·0%	High chloroquine concentrations
Garg et al (1995)	Report	India	Bombay	2	..	..	High chloroquine concentrations but slow clearance
Lee et al (2009)	Report	South Korea	Seoul	2	..	..	Prolonged time to parasite clearance despite high chloroquine concentration
Schuurkamp et al (1989)	Report	PNG	Olsobip	3	..	..	Still positive at day 7
Shah et al (2008)	Report	India	Mumbai	1	..	..	Still positive on day 3
Singh et al (2002)	Report	India	Jabalpur	1	..	..	Still positive on day 4

PNG=Papua New Guinea. ··=no data. References for all studies are shown in the appendix.

## Discussion

The treatment of *P vivax* malaria has changed little in the past 60 years. In most areas chloroquine plus primaquine is the first-line treatment, but this status quo is increasingly threatened by the emergence and spread of chloroquine-resistant *P vivax*. Unequivocal evidence now exists of high-grade chloroquine resistance on the islands of New Guinea and Indonesia,^18^, ^19^ and evidence is accumulating for declining chloroquine efficacy in many other *P vivax*-endemic areas. The extent of this threat is unclear because primaquine has intrinsic blood-stage activity, which could mask low-level chloroquine resistance, and modest reductions in therapeutic efficacy can be either masked or accentuated by various methodological issues inherent in the study designs applied. Our meta-analysis draws attention to this heterogeneity and identifies several key factors contributing to the geospatial uncertainty in *P vivax* drug efficacy (table 3).

**Table 3:** Factors contributing to the geospatial uncertainty in *Plasmodium vivax* drug efficacy

	**Explanation**	**Recommendation**
**Chloroquine sensitivity**		
Enrolment of patients without clinical disease	Host immunity in asymptomatic patients enrolled from cross-sectional surveys might enable clearance of parasitaemia even after partly effective drug treatment	Restrict efficacy trials to patients presenting with clinical disease
Co-administration of early primaquine	Early primaquine has schizonticidal activity that can increase parasite clearance and prevent recrudescent infections	Primaquine treatment should be delayed until the end of the follow-up
Short duration of follow-up	Early evidence of resistance is shown by late recrudescence	Patients should be followed up for a minimum of 28 days
**Incorrect diagnosis of chloroquine resistance**		
Incomplete treatment course	Poor patient adherence	Supervision of drug treatment
Dose of chloroquine too low	Prescription of inadequate mg/kg dose	Documentation of exact dose of drug administered
Poor absorption of drug	Either from poor quality drug or reduced gastrointestinal absorption	Measurement of drug blood concentrations on day 7 and the day of parasite recurrence
Poor drug quality	Faulty product	Confirmation of adequate drug concentrations, pharmacological assessment of study drugs and purchase only from certified, trusted producers

A protocol template for researchers to adapt for the study of *P vivax* antimalarial efficacy is available online.^15^

Decreasing antimalarial efficacy is shown by the ability of malaria parasites to grow in the presence of adequate bloodstream drug concentrations. At low levels of resistance an initial clinical response occurs, often followed by a return of illness caused by recrudescent parasitaemias (a late treatment failure, or late parasitological failure). The length of the interval from the start of treatment to parasite recrudescence depends on the pharmacology of the initial treatment regimen, the degree of drug resistance, and the level of host immunity.^8^ Increasing drug resistance enables parasite growth in high drug concentrations, which slows parasite clearance and shortens the interval to the first recurrence. In studies with a greater risk of recurrence by day 28 illness tended to recur sooner (*r*_s_=–0·58). Highly resistant parasites continue to grow despite high blood concentrations of the drug, which results in early treatment failure. Our review identified 20 clinical trials and case reports in which this result was documented (table 2).

The epicentre for chloroquine-resistant *P vivax* is on the island of New Guinea, where studies have consistently shown high-grade resistance manifested by early clinical deterioration requiring hospitalisation, by delayed parasite clearance, and by early recurrent parasitaemia.^18^, ^19^, ^20^ Several reports of severe and fatal vivax malaria have been published in the past few years.^12^, ^21^ These reports occur more frequently from poorly resourced endemic regions where populations have poor access to health care and have a complex range of comorbidities. Delays in diagnosis, partially effective treatment regimens, and failure to provide a radical cure are important factors in determination of morbidity and associated mortality of *P vivax,* especially that of severe anaemia in young children.^22^, ^23^

Most *P vivax* clinical trials are done in malaria-endemic regions. Patients might therefore acquire new infections during follow-up. This risk is proportional to the duration of follow-up and the intensity of transmission. Relapses also occur, and again the risk is linked to duration of follow-up, host immunity, and the geographical location of the study. The risk of relapse varies substantially, from 50–80% in equatorial regions to 5–10% in temperate areas, where the first relapse occurs many months after the initial infection.^24^ Molecular genotyping methods can discriminate between genetically homologous and heterologous infections.^25^, ^26^, ^27^, ^28^ Our analysis identified ten studies that had used genotyping in analysis of *P vivax* drug efficacy. However, genotyping cannot discriminate between a recrudescence of the blood-stage infection or a relapse with a homologous strain. Furthermore, as relapse can also be with a heterologous parasite strain, reliable distinction of the causes of recurrent infection within an endemic area is not possible at present.^26^, ^29^ The derived estimates of clinical efficacy for *P vivax* therefore are a combination of blood-stage schizonticidal activity and post-treatment prophylaxis suppressing early relapse or reinfection. The unadjusted risk of *P vivax* recurrence needs to be interpreted in light of the half-life of the treatment regimen, concomitant use of primaquine, the timing of recurrence, available molecular data, schizonticidal drug concentrations, entomological inoculation rates, and the epidemiology of relapse in the study area.

Recurrent parasitaemia within 28 days of chloroquine treatment does not necessarily imply chloroquine resistance. Sensitive parasites that have been exposed to insufficient treatment because of an incorrect weight-adjusted dose, poor quality drug, poor gastrointestinal absorption, or unusual pharmacokinetics can also recrudesce early. The definitive diagnosis of chloroquine resistance therefore requires documentation of adequate drug exposure at the time of recurrence, to confirm the growth of parasites in concentrations of drug above the minimum inhibitory concentration.^16^ However, pharmacokinetic analysis requires blood sampling and storage, access to an accredited laboratory, and the associated expenses. Fewer than a third of *P vivax* clinical studies reported were supported by pharmacokinetic analysis.

In falciparum malaria, the speed of parasite clearance correlates with the risk and timing of recrudescence and the infection's transmission potential.^8^ This correlation has become a key clinical parameter for definition of artemisinin resistance.^30^, ^31^ Our analysis draws attention to the importance of the early parasite response after chloroquine treatment of *P vivax*; delayed parasite clearance (defined by prevalence of microscopy detectable parasitaemia at 48 h) and early recurrence were strongly correlated (*r*_s_=–0·72). Two studies have explored this correlation with individual patient's data. Results of the first, in Papua New Guinea, showed that a *P vivax* parasite reduction ratio of less than 7·4 predicted subsequent treatment failure with 70% sensitivity and 63% specificity.^18^ The second, from Thailand, showed that patients still parasitaemic at 48 h had a four-times increased risk of *P vivax* recurrence by day 28.^32^ The stage of parasites' development and the speed of response were correlated, consistent with in-vitro data suggestive of a substantial stage specificity of action of chloroquine in *P vivax*.^11^, ^33^ Because the proportions of parasites at different stages vary substantially between populations, the clinical consequences of parasite staging as a confounding factor for clinical efficacy monitoring warrants further exploration.

A major advantage of parasite clearance rate as a marker for resistance is that it is not confounded by relapse or reinfection. In this Review, clearance of parasitaemia (by microscopy) in all patients by day 3 or in 95% of patients by day 2 was 100% predictive of chloroquine sensitivity in the study population, as defined by the day 28 recurrence rate. Further studies are needed to investigate early parasite clearance for monitoring of *P vivax* in different endemic settings, and the optimum sampling strategy to define it.

Most national guidelines recommend a combination of chloroquine for rapid blood-stage activity and a 14 day course of primaquine for the radical cure of the hypnozoite stages in *P vivax* malaria. Primaquine is usually started with the initial dose of chloroquine. Primaquine has activity against both blood and liver stages, including against chloroquine-resistant strains.^34^, ^35^ Primaquine might therefore provide a good therapeutic response even when combined with a compromised schizonticidal partner drug. Our analysis identified 14 studies that compared chloroquine monotherapy with chloroquine plus primaquine. Patients treated with the combination regimen had a significantly lower risk of recurrence by day 28 (OR 0·27) than did patients given monotherapy. Although this difference was most notable for patients in the Indonesian studies with low chloroquine efficacy, the same decreases were apparent in all but two studies, both of which gave very low dose primaquine regimens (figure 2). Almost a quarter of the studies assessing chloroquine efficacy also treated patients with the early administration of primaquine, and nearly all of these studies reported low rates of recurrent parasitaemia at day 28. Studies with early primaquine are therefore unhelpful in predicting chloroquine resistance. These findings emphasise the additional value of primaquine and the importance in therapeutic assessments (as opposed to routine treatment) of delaying, when possible, the administration of primaquine until the end of treatment, if the primary objective is to detect underlying chloroquine efficacy.

Drug-resistant plasmodia often occur in areas of high drug pressure and low parasite prevalence. When incidence is low, recruitment of patients to studies might be slow. Some investigators have used active recruitment, seeking out patients with parasitaemia in cross-sectional surveys of target populations. However, patients identified in this manner have significantly lower peripheral parasitaemias compared with those of symptomatic patients presenting to a health-care facility. Since the initial parasitaemia before treatment is a major determinant of therapeutic efficacy, studies enrolling patients through active case detection are likely to underestimate resistance. Individuals with low-grade parasitaemias probably have sufficient immunity to suppress symptomatic disease, thereby reducing the risk of recrudescence even from drug-resistant parasites. Antimalarial efficacy studies should aim to document the clinical response in symptomatic patients. Although a high risk of recurrence in patients recruited from surveys can draw attention to areas of potential drug-resistant *P vivax*, the absence of recurrences from such studies should not be held as evidence of drug sensitivity.

Studies of chloroquine efficacy for *P vivax* were categorised according to the risk of recurrence at day 28, the 95% CI on this estimate, the documentation of blood drug concentrations, the enrolment of patients with symptomatic disease, and the early co-administration of primaquine. Our definitions were conservative, with data excluded from almost 40% of studies that we thought could be confounded. Almost half of the informative studies suggested evidence of reduced chloroquine efficacy. 15 of these studies and five case reports documented evidence of early treatment failure (table 2).

The geographical extent of chloroquine-resistant *P vivax* suggests that chloroquine resistance has emerged or spread in many *P vivax*-endemic countries (figure 3). These individual reports warrant closer scrutiny of the raw data, review of quality-control procedures, and repetition, preferably in randomised control trials with alternative treatment regimens. The epicentres of chloroquine-resistance are the eastern provinces of Indonesia, although reports of reduced susceptibility are apparent across the archipelago. In the past 5 years, convincing evidence has been reported of chloroquine resistance *P vivax* from Thailand,^32^ Cambodia,^36^ Ethiopia,^37^, ^38^ and South America.^39^ By contrast, the reports from the Indian subcontinent (India, Afghanistan, and Pakistan) are mostly reassuring. Several reports have suggested either low-grade chloroquine resistance or sporadic reports of early treatment failure;^40^, ^41^ however, these reports have usually not been substantiated on further investigation.

The widespread emergence and spread of chloroquine resistance should cause substantial concern. The combination of chloroquine plus primaquine retains good efficacy in areas with moderate levels of chloroquine resistance, and is the default in most *P vivax* endemic countries. However, in practice, clinicians are often reluctant to prescribe primaquine because of fears of haemolysis.^42^ Furthermore, even when primaquine is prescribed, patients might not adhere to the standard 14 day course. Alternative treatment regimens are available. Most of the artemisinin-based combination therapies have proven efficacy against chloroquine-resistant strains of *P vivax*.^20^, ^43^ Although more costly, a universal policy of artemisinin-based combination therapies in areas co-endemic for both *P falciparum* and *P vivax* has many advantages. The rationale for the use of artemisinin-based combination therapies for all malaria infections is compelling in areas where chloroquine resistance is established.^44^

*P vivax* continues to inflict a substantial burden on the malaria-endemic world with the morbidity and mortality related to its propensity to cause recurrent infections. Frequent recurrences of vivax malaria result from the use of partially effective blood-stage treatment and our poor ability to achieve radical cure safely.^45^ In this context, enhanced monitoring of decreasing drug efficacy for *P vivax* malaria is essential. Enhanced monitoring will require standardised methods and novel approaches for more precise quantification of drug efficacy. A tendency exists to assume that present antimalarial treatment regimens continue to retain efficacy long after evidence of decreasing antimalarial activity has begun to emerge. Inadequate surveillance methods compounded by complacency have delayed the detection and containment of chloroquine-resistant *P falciparum,* with devastating public health consequences. If a repetition of history is to be avoided, then the threat of chloroquine-resistant *P vivax* needs to be acknowledged and greater resources applied to develop standardised and validated methods for its detection, characterisation, and containment.
