# Supplementary material for: Global extent of chloroquine-resistant Plasmodium vivax: a systematic review and meta-analysis
Source: Lancet Infect Dis. 2014 Sep 8;14(10):982–91. doi: 10.1016/S1473-3099(14)70855-2 (PMC4178238; doi:10.1016/S1473-3099(14)70855-2)
Supplement: Supplementary appendix [file mmc1.pdf]

THE LANCET Infectious Diseases

Supplementary webappendix

This webappendix formed part of the original submission and has been peer reviewed. We post it as supplied by the authors.

Supplement to: Price RN, von Seidlein L, Valecha N, Nosten F, Baird JK, White NJ. Global extent of chloroquine-resistant Plasmodium vivax: a systematic review and meta-analysis. *Lancet Infect Dis* 2014; published online Sept 9. http://dx.doi.org/10.1016/S1473-3099(14)70855-2.

Clinical Trial References

1. Abdallah TM, Ali AA, Bakri M, Gasim GI, Musa IR, Adam I. Efficacy of artemether-lumefantrine as a treatment for uncomplicated Plasmodium vivax malaria in eastern Sudan. Malaria journal. 2012;11:404.

2. Adak T, Valecha N, Sharma VP. Plasmodium vivax polymorphism in a clinical drug trial. Clinical and diagnostic laboratory immunology. 2001 Sep;8(5):891-4.

3. Alcantara AK, Uylangco CV, Sangalang RP, Cross JH. A comparative clinical study of mefloquine and chloroquine in the treatment of vivax malaria. The Southeast Asian journal of tropical medicine and public health. 1985 Dec;16(4):534-8.

4. Anez A, Navarro-Costa D, Yucra O, Garnica C, Melgar V, Moscoso M, et al. [Therapeutic response of Plasmodium vivax to chloroquine in Bolivia]. Biomedica : revista del Instituto Nacional de Salud. 2012 Oct-Dec;32(4):527-35.

5. Awab GR, Pukrittayakamee S, Imwong M, Dondorp AM, Woodrow CJ, Lee SJ, et al. Dihydroartemisinin-piperaquine versus chloroquine to treat vivax malaria in Afghanistan: an open randomized, non-inferiority, trial. Malaria journal. 2010;9:105.

6. Baird JK, Basri H, Subianto B, Fryauff DJ, McElroy PD, Leksana B, et al. Treatment of chloroquine-resistant Plasmodium vivax with chloroquine and primaquine or halofantrine. The Journal of infectious diseases. 1995 Jun;171(6):1678-82.

7. Baird JK, Caneta-Miguel E, Masbar S, Bustos DG, Abrenica JA, Layawen AV, et al. Survey of resistance to chloroquine of falciparum and vivax malaria in Palawan, The Philippines. Transactions of the Royal Society of Tropical Medicine and Hygiene. 1996 Jul-Aug;90(4):413-4.

8. Baird JK, Sismadi P, Masbar S, Leksana B, Sekartuti, Ramzan A, et al. Chloroquine sensitive Plasmodium falciparum and P. vivax in central Java, Indonesia. Transactions of the Royal Society of Tropical Medicine and Hygiene. 1996 Jul-Aug;90(4):412-3.

9. Baird JK, Sustriayu Nalim MF, Basri H, Masbar S, Leksana B, Tjitra E, et al. Survey of resistance to chloroquine by Plasmodium vivax in Indonesia. Transactions of the Royal Society of Tropical Medicine and Hygiene. 1996 Jul-Aug;90(4):409-11.

10. Baird JK, Wiady I, Fryauff DJ, Sutanihardja MA, Leksana B, Widjaya H, et al. In vivo resistance to chloroquine by Plasmodium vivax and Plasmodium falciparum at Nabire, Irian Jaya, Indonesia. The American journal of tropical medicine and hygiene. 1997 Jun;56(6):627-31.

11. Baird JK, Tiwari T, Martin GJ, Tamminga CL, Prout TM, Tjaden J, et al. Chloroquine for the treatment of uncomplicated malaria in Guyana. Annals of tropical medicine and parasitology. 2002 Jun;96(4):339-48.

12. Barnadas C, Ratsimbasoa A, Tichit M, Bouchier C, Jahevitra M, Picot S, et al. Plasmodium vivax resistance to chloroquine in Madagascar: clinical efficacy and polymorphisms in pvmdr1 and pvcrt-o genes. Antimicrobial agents and chemotherapy. 2008 Dec;52(12):4233-40.

13. Barnadas C, Tichit M, Bouchier C, Ratsimbasoa A, Randrianasolo L, Raherinjafy R, et al. Plasmodium vivax dhfr and dhps mutations in isolates from Madagascar and therapeutic response to sulphadoxine-pyrimethamine. Malaria journal. 2008;7:35.

14. Castillo CM, Osorio LE, Palma GI. Assessment of therapeutic response of Plasmodium vivax and Plasmodium falciparum to chloroquine in a Malaria transmission free area in Colombia. Memorias do Instituto Oswaldo Cruz. 2002 Jun;97(4):559-62.

15. Congpuon K, Satimai W, Sujariyakul A, Intanakom S, Harnpitakpong W, Pranuth Y, et al. In vivo sensitivity monitoring of chloroquine for the treatment of uncomplicated vivax malaria in four bordered provinces of Thailand during 2009-2010. Journal of vector borne diseases. 2011 Dec;48(4):190-6.

16. Congpuong K, Na-Bangchang K, Thimasarn K, Tasanor U, Wernsdorfer WH. Sensitivity of Plasmodium vivax to chloroquine in Sa Kaeo Province, Thailand. Acta tropica. 2002 Aug;83(2):117-21.

17. Dao NV, Cuong BT, Ngoa ND, Thuy le TT, The ND, Duy DN, et al. Vivax malaria: preliminary observations following a shorter course of treatment with artesunate plus primaquine. Transactions of the Royal Society of Tropical Medicine and Hygiene. 2007 Jun;101(6):534-9.

18. Darlow B, Vrbova H, Gibney S, Jolley D, Stace J, Alpers M. Sulfadoxine-pyrimethamine for the treatment of acute malaria in children of Papua New Guinea. II. Plasmodium vivax. The American journal of tropical medicine and hygiene. 1982 Jan;31(1):10-3.

19. de Santana Filho FS, Arcanjo AR, Chehuan YM, Costa MR, Martinez-Espinosa FE, Vieira JL, et al. Chloroquine-resistant Plasmodium vivax, Brazilian Amazon. Emerging infectious diseases. 2007 Jul;13(7):1125-6.

20. Dilmec F, Kurcer MA, Akkafa F, Simsek Z. Monitoring of failure of chloroquine treatment for Plasmodium vivax using polymerase chain reaction in Sanliurfa province, Turkey. Parasitology research. 2010 Mar;106(4):783-8.

21. Dixon KE, Pitaktong U, Phintuyothin P. A clinical trial of mefloquine in the treatment of Plasmodium vivax malaria. The American journal of tropical medicine and hygiene. 1985 May;34(3):435-7.

22. Dunne MW, Singh N, Shukla M, Valecha N, Bhattacharyya PC, Patel K, et al. A double-blind, randomized study of azithromycin compared to chloroquine for the treatment of Plasmodium vivax malaria in India. The American journal of tropical medicine and hygiene. 2005 Dec;73(6):1108-11.

23. Ebisawa I, Ohara H. A combination of sulfamonomethoxine and pyrimethamine versus other drugs for the treatment of malaria. The Japanese journal of experimental medicine. 1986 Oct;56(5):213-9.

24. Eibach D, Ceron N, Krishnalall K, Carter K, Bonnot G, Bienvenu AL, et al. Therapeutic efficacy of artemether-lumefantrine for Plasmodium vivax infections in a prospective study in Guyana. Malaria journal. 2012;11:347.

25. Fryauff DJ, Baird JK, Candradikusuma D, Masbar S, Sutamihardja MA, Leksana B, et al. Survey of in vivo sensitivity to chloroquine by Plasmodium falciparum and P. vivax in Lombok, Indonesia. The American journal of tropical medicine and hygiene. 1997 Feb;56(2):241-4.

26. Fryauff DJ, Soekartono, Tuti S, Leksana B, Suradi, Tandayu S, et al. Survey of resistance in vivo to chloroquine of Plasmodium falciparum and P. vivax in North Sulawesi, Indonesia. Transactions of the Royal Society of Tropical Medicine and Hygiene. 1998 Jan-Feb;92(1):82-3.

27. Fryauff DJ, Sumawinata I, Purnomo, Richie TL, Tjitra E, Bangs MJ, et al. In vivo responses to antimalarials by Plasmodium falciparum and Plasmodium vivax from isolated Gag Island off northwest Irian Jaya, Indonesia. The American journal of tropical medicine and hygiene. 1999 Apr;60(4):542-6.

28. Fryauff DJ, Tuti S, Mardi A, Masbar S, Patipelohi R, Leksana B, et al. Chloroquine-resistant Plasmodium vivax in transmigration settlements of West Kalimantan, Indonesia. The American journal of tropical medicine and hygiene. 1998 Oct;59(4):513-8.

29. Fryauff DJ, Leksana B, Masbar S, Wiady I, Sismadi P, Susanti AI, et al. The drug sensitivity and transmission dynamics of human malaria on Nias Island, North Sumatra, Indonesia. Annals of tropical medicine and parasitology. 2002 Jul;96(5):447-62.

30. Ganguly S, Saha P, Guha SK, Das S, Bera DK, Biswas A, et al. In vivo therapeutic efficacy of chloroquine alone or in combination with primaquine against vivax malaria in Kolkata, West Bengal, India, and polymorphism in pvmdr1 and pvcrt-o genes. Antimicrobial agents and chemotherapy. 2013 Mar;57(3):1246-51.

31. Genton B, Baea K, Lorry K, Ginny M, Wines B, Alpers MP. Parasitological and clinical efficacy of standard treatment regimens against Plasmodium falciparum, P. vivax and P. malariae in Papua New Guinea. Papua and New Guinea medical journal. 2005 Sep-Dec;48(3-4):141-50.

32. Gogtay NJ, Desai S, Kamtekar KD, Kadam VS, Dalvi SS, Kshirsagar NA. Efficacies of 5- and 14-day primaquine regimens in the prevention of relapses in Plasmodium vivax infections. Annals of tropical medicine and parasitology. 1999 Dec;93(8):809-12.

33. Guthmann JP, Pittet A, Lesage A, Imwong M, Lindegardh N, Min Lwin M, et al. Plasmodium vivax resistance to chloroquine in Dawei, southern Myanmar. Tropical medicine & international health : TM & IH. 2008 Jan;13(1):91-8.

34. Hamedi Y, Nateghpour M, Tan-ariya P, Tiensuwan M, Silachamroon U, Looareesuwan S. Plasmodium vivax malaria in Southeast Iran in 1999-2001: establishing the response to chloroquine in vitro and in vivo. The Southeast Asian journal of tropical medicine and public health. 2002 Sep;33(3):512-8.

35. Hamedi Y, Safa O, Zare S, Tan-ariya P, Kojima S, Looareesuwan S. Therapeutic efficacy of artesunate in Plasmodium vivax malaria in Thailand. The Southeast Asian journal of tropical medicine and public health. 2004 Sep;35(3):570-4.

36. Hapuarachchi HA, Dayanath MY, Abeysundara S, Bandara KB, Abeyewickreme W, de Silva NR. Chloroquine resistant falciparum malaria among security forces personnel in the Northern Province of Sri Lanka. The Ceylon medical journal. 2004 Jun;49(2):47-51.

37. Harinasuta T, Bunnag D, Lasserre R, Leimer R, Vinijanont S. Trials of mefloquine in vivax and of mefloquine plus 'fansidar' in falciparum malaria. Lancet. 1985 Apr 20;1(8434):885-8.

38. Hasugian AR, Purba HL, Kenangalem E, Wuwung RM, Ebsworth EP, Maristela R, et al. Dihydroartemisinin-piperaquine versus artesunate-amodiaquine: superior efficacy and posttreatment prophylaxis against multidrug-resistant Plasmodium falciparum and Plasmodium vivax malaria. Clinical infectious diseases : an official publication of the Infectious Diseases Society of America. 2007 Apr 15;44(8):1067-74.

39. Hasugian AR, Tjitra E, Ratcliff A, Siswantoro H, Kenangalem E, Wuwung RM, et al. In vivo and in vitro efficacy of amodiaquine monotherapy for treatment of infection by chloroquine-resistant Plasmodium vivax. Antimicrobial agents and chemotherapy. 2009 Mar;53(3):1094-9.

40. Heidari A, Keshavarz H, Shojaee S, Raeisi A, Dittrich S. In vivo Susceptibility of Plasmodium vivax to Chloroquine in Southeastern Iran. Iranian journal of parasitology. 2012;7(2):8-14.

41. Hwang J, Alemayehu BH, Reithinger R, Tekleyohannes SG, Takele T, Birhanu SG, et al. In vivo efficacy of artemether-lumefantrine and chloroquine against Plasmodium vivax: a randomized open label trial in central Ethiopia. PloS one. 2013;8(5):e63433.

42. Jagota SC. Halofantrine in the treatment of acute malaria: a multi-centre study in 268 patients. Current medical research and opinion. 1993;13(3):140-4.

43. Kaneko A, Bergqvist Y, Takechi M, Kalkoa M, Kaneko O, Kobayakawa T, et al. Intrinsic efficacy of proguanil against falciparum and vivax malaria independent of the metabolite cycloguanil. The Journal of infectious diseases. 1999 Apr;179(4):974-9.

44. Karunajeewa HA, Mueller I, Senn M, Lin E, Law I, Gomorrai PS, et al. A trial of combination antimalarial therapies in children from Papua New Guinea. The New England journal of medicine. 2008 Dec 11;359(24):2545-57.

45. Ketema T, Bacha K, Birhanu T, Petros B. Chloroquine-resistant Plasmodium vivax malaria in Serbo town, Jimma zone, south-west Ethiopia. Malaria journal. 2009;8:177.

46. Ketema T, Getahun K, Bacha K. Therapeutic efficacy of chloroquine for treatment of Plasmodium vivax malaria cases in Halaba district, South Ethiopia. Parasites & vectors. 2011;4:46.

47. Khan MZ, Isani Z, Ahmed TM, Zafar AB, Gilal N, Maqbool S, et al. Efficacy and safety of halofantrine in Pakistani children and adults with malaria caused by P. falciparum and P. vivax. The Southeast Asian journal of tropical medicine and public health. 2006 Jul;37(4):613-8.

48. Kinzer MH, Chand K, Basri H, Lederman ER, Susanti AI, Elyazar I, et al. Active case detection, treatment of falciparum malaria with combined chloroquine and sulphadoxine/pyrimethamine and vivax malaria with chloroquine and molecular markers of anti-malarial resistance in the Republic of Vanuatu. Malaria journal. 2010;9:89.

49. Kolaczinski K, Durrani N, Rahim S, Rowland M. Sulfadoxine-pyrimethamine plus artesunate compared with chloroquine for the treatment of vivax malaria in areas co-endemic for Plasmodium falciparum and P. vivax: a randomised non-inferiority trial in eastern Afghanistan. Transactions of the Royal Society of Tropical Medicine and Hygiene. 2007 Nov;101(11):1081-7.

50. Krudsood S, Tangpukdee N, Muangnoicharoen S, Thanachartwet V, Luplertlop N, Srivilairit S, et al. Clinical efficacy of chloroquine versus artemether-lumefantrine for Plasmodium vivax treatment in Thailand. The Korean journal of parasitology. 2007 Jun;45(2):111-4.

51. Kurcer MA, Simsek Z, Zeyrek FY, Atay S, Celik H, Kat I, et al. Efficacy of chloroquine in the treatment of Plasmodium vivax malaria in Turkey. Annals of tropical medicine and parasitology. 2004 Jul;98(5):447-51.

52. Kurcer MA, Simsek Z, Kurcer Z. The decreasing efficacy of chloroquine in the treatment of Plasmodium vivax malaria, in Sanliurfa, south-eastern Turkey. Annals of tropical medicine and parasitology. 2006 Mar;100(2):109-13.

53. Lacy MD, Maguire JD, Barcus MJ, Ling J, Bangs MJ, Gramzinski R, et al. Atovaquone/proguanil therapy for Plasmodium falciparum and Plasmodium vivax malaria in Indonesians who lack clinical immunity. Clinical infectious diseases : an official publication of the Infectious Diseases Society of America. 2002 Nov 1;35(9):e92-5.

54. Lal H. A comparative trial of oral chloroquine and oral co-trimoxazole in vivax malaria in children. The American journal of tropical medicine and hygiene. 1982 May;31(3 Pt 1):438-40.

55. Leang R, Barrette A, Bouth DM, Menard D, Abdur R, Duong S, et al. Efficacy of dihydroartemisinin-piperaquine for treatment of uncomplicated Plasmodium falciparum and Plasmodium vivax in Cambodia, 2008 to 2010. Antimicrobial agents and chemotherapy. 2013 Feb;57(2):818-26.

56. Lee SW, Lee M, Lee DD, Kim C, Kim YJ, Kim JY, et al. Biological resistance of hydroxychloroquine for Plasmodium vivax malaria in the Republic of Korea. The American journal of tropical medicine and hygiene. 2009 Oct;81(4):600-4.

57. Leslie T, Mayan I, Mohammed N, Erasmus P, Kolaczinski J, Whitty CJ, et al. A randomised trial of an eight-week, once weekly primaquine regimen to prevent relapse of plasmodium vivax in Northwest Frontier Province, Pakistan. PloS one. 2008;3(8):e2861.

58. Leslie T, Mayan MI, Hasan MA, Safi MH, Klinkenberg E, Whitty CJ, et al. Sulfadoxine-pyrimethamine, chlorproguanil-dapsone, or chloroquine for the treatment of Plasmodium vivax malaria in Afghanistan and Pakistan: a randomized controlled trial. JAMA : the journal of the American Medical Association. 2007 May 23;297(20):2201-9.

59. Li GQ, Guo XB, Fu LC, Jian HX, Wang XH. Clinical trials of artemisinin and its derivatives in the treatment of malaria in China. Transactions of the Royal Society of Tropical Medicine and Hygiene. 1994 Jun;88 Suppl 1:S5-6.

60. Lim CS, Kim YK, Lee KN, Kim MJ, Kim KH, Kim DS, et al. Response to chloroquine of Plasmodium vivax among South Korean soldiers. Annals of tropical medicine and parasitology. 1999 Sep;93(6):565-8.

61. Liu H, Yang HL, Xu JW, Wang JZ, Nie RH, Li CF. Artemisinin-naphthoquine combination versus chloroquine-primaquine to treat vivax malaria: an open-label randomized and non-inferiority trial in Yunnan Province, China. Malaria journal. 2013;12:409.

62. Llanos-Cuentas A, Lacerda MV, Rueangweerayut R, Krudsood S, Gupta SK, Kochar SK, et al. Tafenoquine plus chloroquine for the treatment and relapse prevention of Plasmodium vivax malaria (DETECTIVE): a multicentre, double-blind, randomised, phase 2b dose-selection study. Lancet. 2014 Mar 22;383(9922):1049-58.

63. Lon C, Manning JE, Vanachayangkul P, So M, Sea D, Se Y, et al. Efficacy of two versus three-day regimens of dihydroartemisinin-piperaquine for uncomplicated malaria in military personnel in northern Cambodia: an open-label randomized trial. PloS one. 2014;9(3):e93138.

64. Looareesuwan S, Viravan C, Webster HK, Kyle DE, Hutchinson DB, Canfield CJ. Clinical studies of atovaquone, alone or in combination with other antimalarial drugs, for treatment of acute uncomplicated malaria in Thailand. The American journal of tropical medicine and hygiene. 1996 Jan;54(1):62-6.

65. Looareesuwan S, Wilairatana P, Glanarongran R, Indravijit KA, Supeeranontha L, Chinnapha S, et al. Atovaquone and proguanil hydrochloride followed by primaquine for treatment of Plasmodium vivax malaria in Thailand. Transactions of the Royal Society of Tropical Medicine and Hygiene. 1999 Nov-Dec;93(6):637-40.

66. Looareesuwan S, Wilairatana P, Krudsood S, Treeprasertsuk S, Singhasivanon P, Bussaratid V, et al. Chloroquine sensitivity of Plasmodium vivax in Thailand. Annals of tropical medicine and parasitology. 1999 Apr;93(3):225-30.

67. Luxemburger C, van Vugt M, Jonathan S, McGready R, Looareesuwan S, White NJ, et al. Treatment of vivax malaria on the western border of Thailand. Transactions of the Royal Society of Tropical Medicine and Hygiene. 1999 Jul-Aug;93(4):433-8.

68. Machado RL, de Figuereido Filho AF, Calvosa VS, Figueredo MC, Nascimento JM, Povoa MM. Correlation between Plasmodium vivax variants in Belem, Para State, Brazil and symptoms and clearance of parasitaemia. The Brazilian journal of infectious diseases : an official publication of the Brazilian Society of Infectious Diseases. 2003 Jun;7(3):175-7.

69. Maguire JD, Krisin, Marwoto H, Richie TL, Fryauff DJ, Baird JK. Mefloquine is highly efficacious against chloroquine-resistant Plasmodium vivax malaria and Plasmodium falciparum malaria in Papua, Indonesia. Clinical infectious diseases : an official publication of the Infectious Diseases Society of America. 2006 Apr 15;42(8):1067-72.

70. Maguire JD, Lacy MD, Sururi, Sismadi P, Krisin, Wiady I, et al. Chloroquine or sulfadoxine-pyrimethamine for the treatment of uncomplicated, Plasmodium falciparum malaria during an epidemic in Central Java, Indonesia. Annals of tropical medicine and parasitology. 2002 Oct;96(7):655-68.

71. Marfurt J, Mueller I, Sie A, Maku P, Goroti M, Reeder JC, et al. Low efficacy of amodiaquine or chloroquine plus sulfadoxine-pyrimethamine against Plasmodium falciparum and P. vivax malaria in Papua New Guinea. The American journal of tropical medicine and hygiene. 2007 Nov;77(5):947-54.

72. Marlar T, Myat Phone K, Aye Yu S, Khaing Khaing G, Ma S, Myint O. Development of resistance to chloroquine by Plasmodium vivax in Myanmar. Transactions of the Royal Society of Tropical Medicine and Hygiene. 1995 May-Jun;89(3):307-8.

73. Marques MM, Costa MR, Santana Filho FS, Vieira JL, Nascimento MT, Brasil LW, et al. Plasmodium vivax chloroquine resistance and anemia in the western Brazilian Amazon. Antimicrobial agents and chemotherapy. 2014;58(1):342-7.

74. McGready R, Thwai KL, Cho T, Samuel, Looareesuwan S, White NJ, et al. The effects of quinine and chloroquine antimalarial treatments in the first trimester of pregnancy. Transactions of the Royal Society of Tropical Medicine and Hygiene. 2002 Mar-Apr;96(2):180-4.

75. Mishra N, Singh JP, Srivastava B, Arora U, Shah NK, Ghosh SK, et al. Monitoring antimalarial drug resistance in India via sentinel sites: outcomes and risk factors for treatment failure, 2009-2010. Bulletin of the World Health Organization. 2012 Dec 1;90(12):895-904.

76. Mohapatra MK, Padhiary KN, Mishra DP, Sethy G. Atypical manifestations of Plasmodium vivax malaria. Indian journal of malariology. 2002 Mar-Jun;39(1-2):18-25.

77. Muhamad P, Ruengweerayut R, Chacharoenkul W, Rungsihirunrat K, Na-Bangchang K. Monitoring of clinical efficacy and in vitro sensitivity of Plasmodium vivax to chloroquine in area along Thai Myanmar border during 2009-2010. Malaria journal. 2011;10(1):44.

78. Murphy GS, Basri H, Purnomo, Andersen EM, Bangs MJ, Mount DL, et al. Vivax malaria resistant to treatment and prophylaxis with chloroquine. Lancet. 1993 Jan 9;341(8837):96-100.

79. Nandy A, Addy M, Maji AK, Bandyopadhyay AK. Monitoring the chloroquine sensitivity of Plasmodium vivax from Calcutta and Orissa, India. Annals of tropical medicine and parasitology. 2003 Apr;97(3):215-20.

80. Nateghpour M, Sayadzadeh SA, Edrissan GH, Raeisi A, Jahantigh A, Motevalli-Haghi A, et al. Evaluation of Sensitivity of Plasmodium vivax to Chloroquine. Iranian J Publ Health. 2007;36(3):60-3.

81. Nguyen DS, Dao BH, Nguyen PD, Nguyen VH, Le NB, Mai VS, et al. Treatment of malaria in Vietnam with oral artemisinin. The American journal of tropical medicine and hygiene. 1993 Mar;48(3):398-402.

82. Osorio L, Perez Ldel P, Gonzalez IJ. [Assessment of the efficacy of antimalarial drugs in Tarapaca, in the Colombian Amazon basin]. Biomedica : revista del Instituto Nacional de Salud. 2007 Mar;27(1):133-40.

83. Phan GT, de Vries PJ, Tran BQ, Le HQ, Nguyen NV, Nguyen TV, et al. Artemisinin or chloroquine for blood stage Plasmodium vivax malaria in Vietnam. Tropical medicine & international health : TM & IH. 2002 Oct;7(10):858-64.

84. Phyo AP, Lwin KM, Price RN, Ashley EA, Russell B, Sriprawat K, et al. Dihydroartemisinin-piperaquine versus chloroquine in the treatment of Plasmodium vivax malaria in Thailand: a randomized controlled trial. Clinical infectious diseases : an official publication of the Infectious Diseases Society of America. 2011 Nov;53(10):977-84.

85. Pinto AY, Azevedo CH, da Silva JB, de Souza JM. Assessment of chloroquine single dose treatment of malaria due to Plasmodium vivax in Brazilian Amazon. Revista do Instituto de Medicina Tropical de Sao Paulo. 2003 Nov-Dec;45(6):327-31.

86. Poravuth Y, Socheat D, Rueangweerayut R, Uthaisin C, Pyae Phyo A, Valecha N, et al. Pyronaridine-artesunate versus chloroquine in patients with acute Plasmodium vivax malaria: a randomized, double-blind, non-inferiority trial. PloS one. 2011;6(1):e14501.

87. Potkar CN, Kshirsagar NA, Kathuria R. Resurgence of malaria and drug resistance in plasmodium falciparum and plasmodium vivax species in Bombay. The Journal of the Association of Physicians of India. 1995 May;43(5):336-8.

88. Pukrittayakamee S, Chantra A, Simpson JA, Vanijanonta S, Clemens R, Looareesuwan S, et al. Therapeutic responses to different antimalarial drugs in vivax malaria. Antimicrobial agents and chemotherapy. 2000 Jun;44(6):1680-5.

89. Pukrittayakamee S, Clemens R, Chantra A, Nontprasert A, Luknam T, Looareesuwan S, et al. Therapeutic responses to antibacterial drugs in vivax malaria. Transactions of the Royal Society of Tropical Medicine and Hygiene. 2001 Sep-Oct;95(5):524-8.

90. Pukrittayakamee S, Imwong M, Chotivanich K, Singhasivanon P, Day NP, White NJ. A comparison of two short-course primaquine regimens for the treatment and radical cure of Plasmodium vivax malaria in Thailand. The American journal of tropical medicine and hygiene. 2010 Apr;82(4):542-7.

91. Pukrittayakamee S, Vanijanonta S, Chantra A, Clemens R, White NJ. Blood stage antimalarial efficacy of primaquine in Plasmodium vivax malaria. The Journal of infectious diseases. 1994 Apr;169(4):932-5.

92. Pukrittayakamee S, Viravan C, Charoenlarp P, Yeamput C, Wilson RJ, White NJ. Antimalarial effects of rifampin in Plasmodium vivax malaria. Antimicrobial agents and chemotherapy. 1994 Mar;38(3):511-4.

93. Rajgor DD, Gogtay NJ, Kadam VS, Kamtekar KD, Dalvi SS, Chogle AR, et al. Efficacy of a 14-day primaquine regimen in preventing relapses in patients with Plasmodium vivax malaria in Mumbai,

India. Transactions of the Royal Society of Tropical Medicine and Hygiene. 2003 Jul-Aug;97(4):438-40.

94. Rao KS, Kamalakar KV. Efficacy and safety of halofantrine in acute malaria. The Journal of the Association of Physicians of India. 1993 Aug;41(8):507-8.

95. Ratcliff A, Siswantoro H, Kenangalem E, Maristela R, Wuwung RM, Laihad F, et al. Two fixed-dose artemisinin combinations for drug-resistant falciparum and vivax malaria in Papua, Indonesia: an open-label randomised comparison. Lancet. 2007 Mar 3;369(9563):757-65.

96. Ratcliff A, Siswantoro H, Kenangalem E, Wuwung M, Brockman A, Edstein MD, et al. Therapeutic response of multidrug-resistant Plasmodium falciparum and P. vivax to chloroquine and sulfadoxine-pyrimethamine in southern Papua, Indonesia. Transactions of the Royal Society of Tropical Medicine and Hygiene. 2007 Apr;101(4):351-9.

97. Rios A, Alvarez G, Blair S. [Ten years of chloroquine efficacy for uncomplicated Plasmodium vivax malaria treatment, Turbo, Antioquia, 2002 and 2011]. Biomedica : revista del Instituto Nacional de Salud. 2013 Jul-Sep;33(3):429-38.

98. Rowland M, Durrani N. Randomized controlled trials of 5- and 14-days primaquine therapy against relapses of vivax malaria in an Afghan refugee settlement in Pakistan. Transactions of the Royal Society of Tropical Medicine and Hygiene. 1999 Nov-Dec;93(6):641-3.

99. Ruebush TK, 2nd, Zegarra J, Cairo J, Andersen EM, Green M, Pillai DR, et al. Chloroquine-resistant Plasmodium vivax malaria in Peru. The American journal of tropical medicine and hygiene. 2003 Nov;69(5):548-52.

100. Saravu K, Acharya V, Kumar K, Kumar R. Plasmodium vivax remains responsive to chloroquine with primaquine treatment regimen: a prospective cohort study from tertiary care teaching hospital in southern India. Tropical doctor. 2012 Jul;42(3):163-4.

101. Shalini S, Chaudhuri S, Sutton PL, Mishra N, Srivastava N, David JK, et al. Chloroquine efficacy studies confirm drug susceptibility of Plasmodium vivax in Chennai, India. Malaria journal. 2014;13:129.

102. Silachamroon U, Krudsood S, Treeprasertsuk S, Wilairatana P, Chalearmrult K, Mint HY, et al. Clinical trial of oral artesunate with or without high-dose primaquine for the treatment of vivax malaria in Thailand. The American journal of tropical medicine and hygiene. 2003 Jul;69(1):14-8.

103. Singh RK. Emergence of chloroquine-resistant vivax malaria in south Bihar (India). Transactions of the Royal Society of Tropical Medicine and Hygiene. 2000 May-Jun;94(3):327.

104. Soto J, Toledo J, Gutierrez P, Luzz M, Llinas N, Cedeno N, et al. Plasmodium vivax clinically resistant to chloroquine in Colombia. The American journal of tropical medicine and hygiene. 2001 Aug;65(2):90-3.

105. Srivastava HC, Yadav RS, Joshi H, Valecha N, Mallick PK, Prajapati SK, et al. Therapeutic responses of Plasmodium vivax and P. falciparum to chloroquine, in an area of western India where P. vivax predominates. Annals of tropical medicine and parasitology. 2008 Sep;102(6):471-80.

106. Sumawinata IW, Bernadeta, Leksana B, Sutamihardja A, Purnomo, Subianto B, et al. Very high risk of therapeutic failure with chloroquine for uncomplicated Plasmodium falciparum and P. vivax malaria in Indonesian Papua. The American journal of tropical medicine and hygiene. 2003 Apr;68(4):416-20.

107. Sutanto I, Endawati D, Ling LH, Laihad F, Setiabudy R, Baird JK. Evaluation of chloroquine therapy for vivax and falciparum malaria in southern Sumatra, western Indonesia. Malaria journal. 2010;9:52.

108. Sutanto I, Suprijanto S, Nurhayati, Manoempil P, Baird JK. Resistance to chloroquine by Plasmodium vivax at Alor in the Lesser Sundas Archipelago in eastern Indonesia. The American journal of tropical medicine and hygiene. 2009 Aug;81(2):338-42.

109. Tan-ariya P, Na-Bangchang K, Tin T, Limpaibul L, Brockelman CR, Karbwang J. Clinical response and susceptibility in vitro of Plasmodium vivax to the standard regimen of chloroquine in Thailand. Transactions of the Royal Society of Tropical Medicine and Hygiene. 1995 Jul-Aug;89(4):426-9.

110. Tasanor O, Ruengweerayut R, Sirichaisinthop J, Congpuong K, Wernsdorfer WH, Na-Bangchang K. Clinical-parasitological response and in-vitro sensitivity of Plasmodium vivax to chloroquine and quinine on the western border of Thailand. Transactions of the Royal Society of Tropical Medicine and Hygiene. 2006 May;100(5):410-8.

111. Taylor WR, Doan HN, Nguyen DT, Tran TU, Fryauff DJ, Gomez-Saladin E, et al. Assessing drug sensitivity of Plasmodium vivax to halofantrine or choroquine in southern, central Vietnam using an extended 28-day in vivo test and polymerase chain reaction genotyping. The American journal of tropical medicine and hygiene. 2000 Jun;62(6):693-7.

112. Taylor WR, Widjaja H, Richie TL, Basri H, Ohrt C, Tjitra, et al. Chloroquine/doxycycline combination versus chloroquine alone, and doxycycline alone for the treatment of Plasmodium falciparum and Plasmodium vivax malaria in northeastern Irian Jaya, Indonesia. The American journal of tropical medicine and hygiene. 2001 May-Jun;64(5-6):223-8.

113. Teka H, Petros B, Yamuah L, Tesfaye G, Elhassan I, Muchohi S, et al. Chloroquine-resistant Plasmodium vivax malaria in Debre Zeit, Ethiopia. Malaria journal. 2008;7:220.

114. Tjitra E, Baker J, Suprianto S, Cheng Q, Anstey NM. Therapeutic efficacies of artesunate-sulfadoxine-pyrimethamine and chloroquine-sulfadoxine-pyrimethamine in vivax malaria pilot studies: relationship to Plasmodium vivax dhfr mutations. Antimicrobial agents and chemotherapy. 2002 Dec;46(12):3947-53.

115. Tjitra E, Hasugian AR, Siswantoro H, Prasetyorini B, Ekowatiningsih R, Yusnita EA, et al. Efficacy and safety of artemisinin-naphthoquine versus dihydroartemisinin-piperaquine in adult patients with uncomplicated malaria: a multi-centre study in Indonesia. Malaria journal. 2012;11:153.

116. Trujillo SB, Castano AT, Restrepo ME, Sanchez GA, Fonseca JC. [Adequate clinical and parasitological Plasmodium vivax response to chloroquine in Colombia (Turbo, Antioquia), 2001]. Infectio. 2002;6(1):21-6.

117. Tulu AN, Webber RH, Schellenberg JA, Bradley DJ. Failure of chloroquine treatment for malaria in the highlands of Ethiopia. Transactions of the Royal Society of Tropical Medicine and Hygiene. 1996 Sep-Oct;90(5):556-7.

118. Valecha N, Joshi H, Eapen A, Ravinderan J, Kumar A, Prajapati SK, et al. Therapeutic efficacy of chloroquine in Plasmodium vivax from areas with different epidemiological patterns in India and their Pvdhfr gene mutation pattern. Transactions of the Royal Society of Tropical Medicine and Hygiene. 2006 Sep;100(9):831-7.

119. Valibayov A, Abdullayev F, Mammadov S, Gasimov E, Sabatinelli G, Kondrachine AV, et al. Clinical efficacy of chloroquine followed by primaquine for Plasmodium vivax treatment in Azerbaijan. Acta tropica. 2003 Sep;88(1):99-102.

120. Vijaykadga S, Rojanawatsirivej C, Congpoung K, Wilairatana P, Satimai W, Uaekowitchai C, et al. Assessment of therapeutic efficacy of chloroquine for vivax malaria in Thailand. The Southeast Asian journal of tropical medicine and public health. 2004 Sep;35(3):566-9.

121. Villalobos-Salcedo JM, Tada MS, Kimura E, Menezes MJ, Pereira da Silva LH. In-vivo sensitivity of Plasmodium vivax isolates from Rond nia (western Amazon region, Brazil) to regimens including chloroquine and primaquine. Annals of tropical medicine and parasitology. 2000 Dec;94(8):749-58.

122. Walsh DS, Looareesuwan S, Wilairatana P, Heppner DG, Jr., Tang DB, Brewer TG, et al. Randomized dose-ranging study of the safety and efficacy of WR 238605 (Tafenoquine) in the prevention of relapse of Plasmodium vivax malaria in Thailand. The Journal of infectious diseases. 1999 Oct;180(4):1282-7.

123. Walsh DS, Wilairatana P, Tang DB, Heppner DG, Jr., Brewer TG, Krudsood S, et al. Randomized trial of 3-dose regimens of tafenoquine (WR238605) versus low-dose primaquine for preventing Plasmodium vivax malaria relapse. Clinical infectious diseases : an official publication of the Infectious Diseases Society of America. 2004 Oct 15;39(8):1095-103.

124. Wilairatana P, Silachamroon U, Krudsood S, Singhasivanon P, Treeprasertsuk S, Bussaratid V, et al. Efficacy of primaquine regimens for primaquine-resistant Plasmodium vivax malaria in Thailand. The American journal of tropical medicine and hygiene. 1999 Dec;61(6):973-7.

125. Yadav RS, Ghosh SK. Radical curative efficacy of five-day regimen of primaquine for treatment of Plasmodium vivax malaria in India. The Journal of parasitology. 2002 Oct;88(5):1042-4.

126. Yeramian P, Meshnick SR, Krudsood S, Chalermrut K, Silachamroon U, Tangpukdee N, et al. Efficacy of DB289 in Thai patients with Plasmodium vivax or acute, uncomplicated Plasmodium falciparum infections. The Journal of infectious diseases. 2005 Jul 15;192(2):319-22.

127. Yeshiwondim AK, Tekle AH, Dengela DO, Yohannes AM, Teklehaimanot A. Therapeutic efficacy of chloroquine and chloroquine plus primaquine for the treatment of Plasmodium vivax in Ethiopia. Acta tropica. 2010 Feb;113(2):105-13.

128. Yohannes AM, Teklehaimanot A, Bergqvist Y, Ringwald P. Confirmed vivax resistance to chloroquine and effectiveness of artemether-lumefantrine for the treatment of vivax malaria in Ethiopia. The American journal of tropical medicine and hygiene. 2011 Jan;84(1):137-40.

129. Zhu G, Lu F, Cao J, Zhou H, Liu Y, Han ET, et al. Blood stage of Plasmodium vivax in central China is still susceptible to chloroquine plus primaquine combination therapy. The American journal of tropical medicine and hygiene. 2013 Jul;89(1):184-7.

Case Report References

130. Ahlm C, Wistrom J, Carlsson H. Chloroquine-Resistant Plasmodium vivax Malaria in Borneo. Journal of travel medicine. 1996 Jun 1;3(2):124.

131. Alecrim MdG, Alecrim W, Macedo V. Plasmodium vivax resistance to chloroquine (R2) and mefloquine (R3) in Brazilian Amazon region. Revista da Sociedade Brasileira de Medicina Tropical. 1999 Jan-Feb;32(1):67-8.

132. Arias AE, Corredor A. Low response of Colombian strains of Plasmodium vivax to classical antimalarial therapy. Tropical medicine and parasitology : official organ of Deutsche Tropenmedizinische Gesellschaft and of Deutsche Gesellschaft fur Technische Zusammenarbeit. 1989 Mar;40(1):21-3.

133. Baird JK, Basri H, Purnomo, Bangs MJ, Subianto B, Patchen LC, et al. Resistance to chloroquine by Plasmodium vivax in Irian Jaya, Indonesia. The American journal of tropical medicine and hygiene. 1991 May;44(5):547-52.

134. Barrett JP, Behrens RH. Prophylaxis Failure Against Vivax Malaria in Guyana, South America. Journal of travel medicine. 1996 Mar 1;3(1):60-1.

135. Collignon P. Chloroquine resistance in Plasmodium vivax. The Journal of infectious diseases. 1991 Jul;164(1):222-3.

136. Dua VK, Kar PK, Sharma VP. Chloroquine resistant Plasmodium vivax malaria in India. Tropical medicine & international health : TM & IH. 1996 Dec;1(6):816-9.

137. Garavelli PL, Corti E. Chloroquine resistance in Plasmodium vivax: the first case in Brazil. Transactions of the Royal Society of Tropical Medicine and Hygiene. 1992 Mar-Apr;86(2):128.

138. Garg M, Gopinathan N, Bodhe P, Kshirsagar NA. Vivax malaria resistant to chloroquine: case reports from Bombay. Transactions of the Royal Society of Tropical Medicine and Hygiene. 1995 Nov-Dec;89(6):656-7.

139. Graf PC, Durand S, Alvarez Antonio C, Montalvan C, Galves Montoya M, Green MD, et al. Failure of Supervised Chloroquine and Primaquine Regimen for the Treatment of Plasmodium vivax in the Peruvian Amazon. Malaria research and treatment. 2012;2012:936067.

140. Kshirsagar NA, Gogtay NJ, Rajgor D, Dalvi SS, Wakde M. An unusual case of multidrug-resistant Plasmodium vivax malaria in Mumbai (Bombay), India. Annals of tropical medicine and parasitology. 2000 Mar;94(2):189-90.

141. Lapierre J, Coquelin B, Galal AA, Dupouy-Camet J, Faurant C, Tourte-Schaefer C, et al. [Drug resistance of Plasmodium falciparum and Plasmodium vivax strains in Cambodia (Cardamone Massif). Morphological characteristics of Plasmodium vivax]. Medecine tropicale : revue du Corps de sante colonial. 1984 Oct-Dec;44(4):339-49.

142. Lee KS, Kim TH, Kim ES, Lim HS, Yeom JS, Jun G, et al. Short report: chloroquine-resistant Plasmodium vivax in the Republic of Korea. The American journal of tropical medicine and hygiene. 2009 Feb;80(2):215-7.

143. Lim PL, Mok YJ, Lye DC, Leo YS. Imported chloroquine-resistant Plasmodium vivax in Singapore: case report and literature review. Journal of travel medicine. 2010 Jan-Feb;17(1):69-71.

144. McCullough TJ, Rajabalendaran N, Kirubakaran M, Mollison LC. Chloroquine-resistant Plasmodium vivax from Lombok. The Medical journal of Australia. 1993 Aug 2;159(3):211.

145. Mohan K, Maithani MM. Congenital malaria due to chloroquine-resistant Plasmodium vivax: a case report. Journal of tropical pediatrics. 2010 Dec;56(6):454-5.

146. Myat Phone K, Myint O, Myint L, Thaw Z, Kyin Hla A, Nwe Nwe Y. Emergence of chloroquine-resistant Plasmodium vivax in Myanmar (Burma). Transactions of the Royal Society of Tropical Medicine and Hygiene. 1993 Nov-Dec;87(6):687.

147. Park JW, Jun G, Yeom JS. Plasmodium vivax malaria: status in the Republic of Korea following reemergence. The Korean journal of parasitology. 2009 Oct;47 Suppl:S39-50.

148. Phillips EJ, Keystone JS, Kain KC. Failure of combined chloroquine and high-dose primaquine therapy for Plasmodium vivax malaria acquired in Guyana, South America. Clinical infectious diseases : an official publication of the Infectious Diseases Society of America. 1996 Nov;23(5):1171-3.

149. Rieckmann KH, Davis DR, Hutton DC. Plasmodium vivax resistance to chloroquine? Lancet. 1989 Nov 18;2(8673):1183-4.

150. Rijken MJ, Boel ME, Russell B, Imwong M, Leimanis ML, Phyo AP, et al. Chloroquine resistant vivax malaria in a pregnant woman on the western border of Thailand. Malaria journal. 2011;10:113.

151. Schuurkamp GJ, Spicer PE, Kereu RK, Bulungol PK. A mixed infection of vivax and falciparum malaria apparently resistant to 4-aminoquinoline: a case report. Transactions of the Royal Society of Tropical Medicine and Hygiene. 1989 Sep-Oct;83(5):607-8.

152. Schuurkamp GJ, Spicer PE, Kereu RK, Bulungol PK, Rieckmann KH. Chloroquine-resistant Plasmodium vivax in Papua New Guinea. Transactions of the Royal Society of Tropical Medicine and Hygiene. 1992 Mar-Apr;86(2):121-2.

153. Schwartz IK, Lackritz EM, Patchen LC. Chloroquine-resistant Plasmodium vivax from Indonesia. The New England journal of medicine. 1991 Mar 28;324(13):927.

154. Shah I. Chloroquine resistant vivax malaria in an infant: a report from India. Journal of vector borne diseases. 2008 Jun;45(2):176-7.

155. Singh N, Nagpal AC, Gupta RB. Failure of chloroquine therapy in a splenectomized child infected with Plasmodium vivax. Annals of tropical medicine and parasitology. 2002 Jan;96(1):109-11.

156. Van den Abbeele K, Van den Enden E, Van den Ende J. Combined chloroquine and primaquine resistant Plasmodium vivax malaria in a patient returning from India. Annales de la Societe belge de medecine tropicale. 1995 Mar;75(1):73-4.

157. Whitby M, Wood G, Veenendaal JR, Rieckmann K. Chloroquine-resistant Plasmodium vivax. Lancet. 1989 Dec 9;2(8676):1395.

References of Clinical Trials

First Author	Year	Title	Journal	Vol	Issue	Pages	Acc Number
Abdallah	2012	Efficacy of artemether-lumefantrine as a treatment for uncomplicated Plasmodium vivax malaria in eastern Sudan.	Malaria journal	11	1	404	23217037
Adak	2001	Plasmodium vivax polymorphism in a clinical drug trial	Clin Diagn Lab Immunol	8	5	891-4	11527798
Alcantara	1985	A comparative clinical study of mefloquine and chloroquine in the treatment of vivax malaria.	SE Asian J Trop Med Public Health	16	4	535-539	3915155
Anez	2012	[Therapeutic response of Plasmodium vivax to chloroquine in Bolivia]	Biomedica	32	4	527-35	23715228
Awab	2010	Dihydroartemisinin-piperaquine versus chloroquine to treat vivax malaria in Afghanistan: an open randomized, non-inferiority, trial	Malaria Journal	9	1	105	20409302
Baird	1995	Treatment of chloroquine-resistant Plasmodium vivax with chloroquine and primaquine or halofantrine.	J Infect Dis	171		1678-1682	7769318
Baird	1997	In vivo resistance to chloroquine by Plasmodium vivax and Plasmodium falciparum at Nabire, Irian Jaya, Indonesia.	Am J Trop Med Hyg	56	6	627-631	9230793
Baird	2002	Chloroquine for the treatment of uncomplicated malaria in Guyana.	Ann Trop Med Parasitol	96	4	339-348	12171615
Baird	1996a	Chloroquine sensitive Plasmodium falciparum and P. vivax in central Java, Indonesia	Trans R Soc Trop Med Hyg	90		412-413	8882191
Baird	1996b	Survey of resistance to chloroquine by Plasmodium vivax in Indonesia.	Trans R Soc Trop Med Hyg	90		409-411	8882190
Baird	1996c	Survey of resistance to chloroquine of falciparum and vivax malaria in Palawan, The Philippines.	Trans R Soc Trop Med Hyg	90		413-414	8882192
Barnadas	2008a	Plasmodium vivax resistance to chloroquine in Madagascar: clinical efficacy and polymorphisms in pvmdr1 and pvcrt-o genes	Antimicrob Agents Chemother	52	12	4233-4240	18809933
Barnadas	2008b	Plasmodium vivax dhfr and dhps mutations in isolates from Madagascar and therapeutic response to sulphadoxine-pyrimethamine	Malaria Journal	7	35	doi:10.1186/	18302746
Castillo	2002	Assessment of therapeutic response of Plasmodium vivax and Plasmodium falciparum to chloroquine in a Malaria transmission free area in Colombia.	Mem Inst Oswaldo Cruz	97	4	559-562	12118291
Congpuong	2002	Sensitivity of Plasmodium vivax to chloroquine in Sa Kaeo Province, Thailand.	Acta Trop	83		117-121	12088852
Congpuong	2011	In vivo sensitivity monitoring of chloroquine for the treatment of uncomplicated vivax malaria in four bordered provinces of Thailand during 2009-2010	Journal of vector borne diseases	48	4	190-6	22297279
Dao	2007	Vivax malaria: preliminary observations following a shorter course of treatment with artesunate plus primaquine	Trans R Soc Trop Med Hyg	101		534-539	17368694
Darlow	1982	Sulfadoxine-pyrimethamine for the treatment of acute malaria in children of Papua New Guinea. II. Plasmodium vivax	Am J Trop Med Hyg	31	1	10-13	7036765
de Santana Filho	2007	Chloroquine-resistant Plasmodium vivax, Brazilian Amazon	Emerg Infect Dis	13	7	1125-1127	18214203
Dilmec	2010	Monitoring of failure of chloroquine treatment for *Plasmodium vivax* using polymerase chain reaction in Sanliurfa province, Turkey	Parasitol Res	106		783-788	20140453
Dixon	1985	A clinical trial of mefloquine in the treatment of Plasmodium vivax malaria.	Am J Trop Med Hyg	34	3	435-437	3890575
Dunne	2005	A double blind, randomized study of azithromycin compared to chloroquine for the treatment of Plasmodium Vivax malaria	Am J Trop Med Hyg	73	6	1108-1111	16354821
Ebisawa	1986	A combination of sulfamonomethoxine and pyrimethamine versus other drugs for the treatment of malaria	Jpn J Exp Med	56	5	213-9	3543442
Eibach	2012	Therapeutic efficacy of artemether-lumefantrine for Plasmodium vivax infections in a prospective study in Guyana	Malaria Journal	11		e347	23083017
Fryauff	1997	Survey of in vivo sensitivity to chloroquine by Plasmodium falciparum and P. vivax in Lombok, Indonesia	Am J Trop Med Hyg	56	2	241-244	9080887

References of Clinical Trials

First Author	Year	Title	Journal	Vol	Issue	Pages	Acc Number
Fryauff	1999	In vivo responses to antimalarials by Plasmodium falciparum and Plasmodium vivax from isolated Gag Island off northwest Irian Jaya, Indonesia	Am J Trop Med Hyg	60	4	542-546	10348226
Fryauff	2002	The drug sensitivity and transmission dynamics of human malaria on Nias Island, North Sumatra, Indonesia.	Ann Trop Med Parasitol	96	5	447-462	12194705
Fryauff	1998a	Chloroquine-resistant Plasmodium vivax in transmigration settlements of West Kalimantan, Indonesia.	Am J Trop Med Hyg	59	4	513-518	9790420
Fryauff	1998b	Survey of resistance in vivo to chloroquine of Plasmodium falciparum and P. vivax in North Sulawesi, Indonesia.	Trans R Soc Trop Med Hyg	92		82-83	9692162
Ganguly	2013	In vivo therapeutic efficacy of chloroquine alone or in combination with primaquine in vivax malaria in Kolkata, West Bengal, India and polymorphism in pvmdr1 and pvcrt-0 genes	Antimicrob Agents Chemother	57	3	1246-51	23262997
Genton	2005	Parasitological and clinical efficacy of standard treatment regimens against Plasmodium falciparum, P. vivax and P. malariae in Papua New Guinea.	P N G Med J	48	3	141-150	17212060
Gogtay	1999	Efficacy of 5- and 14- day primaquine regimen in the prevention of relapses in Plasmodium vivax infections	Ann Trop Med Parasitol	93		809-12	10715673
Guthmann	2008	Plasmodium vivax resistance to chloroquine in Dawei, southern Myanmar	Trop Med Int Health	13		91-98	18291007
Hamedi	2002	Plasmodium vivax malaria in Southeast Iran in 1999-2001: establishing the response to chloroquine in vitro and in vivo.	SE Asian J Trop Med Public Health	33	3	512-517	12693585
Hamedi	2004	Therapeutic efficacy of artesunate in Plasmodium vivax malaria in Thailand	SE Asian J Trop Med Public Health	35	3	570-574	15689068
Hapuarachchi	2004	Chloroquine resistant falciparum malaria among security forces personnel in the Northern Province of Sri Lanka.	Ceylon Medical Journal	49	2	47-51	15334798
Harinasuta	1985	Trials of mefloquine in vivax and of mefloquine plus 'fansidar' in falciparum malaria	Lancet	8434		885-888	2858743
Hasugian	2007	Dihydroartemisinin-piperaquine versus artesunate-amodiaquine: superior efficacy and posttreatment prophylaxis against multidrug-resistant Plasmodium falciparum and Plasmodium vivax malaria	Clin Infect Dis	44		1067-74	17366451
Hasugian	2009	In vivo and in vitro efficacy of amodiaquine monotherapy for treatment of infection by chloroquine-resistant Plasmodium vivax	Antimicrob Agents Chemother	53	3	1094-1099	19104023
Heidari	2012	In vivo Susceptibility of Plasmodium vivax to Chloroquine in Southeastern Iran	Iranian Journal of Parasitology	7	2	81-4	23109940
Hwang	2013	In Vivo Efficacy of Artemether-Lumefantrine and Chloroquine against Plasmodium vivax: A Randomized Open Label Trial in Central Ethiopia	PLoS One	8	5	e63433	23717423
Jagota	1993	Halofantrine in the treatment of acute malaria: a multi-centre study in 268 patients	Curr Med Res Opin	13	3	140-144	8222741
Kaneko	1999	Intrinsic efficacy of proguanil against falciparum and vivax malaria independent of the metabolite cycloguanil	J Infect Dis	179		974-979	10068594
Karunajeewa	2008	A trial of combination antimalarial therapies in children from Papua New Guinea.	N Engl J Med	359	24	2545	19064624
Ketema	2009	Chloroquine-resistant Plasmodium vivax malaria in Serbo town, Jimma zone, south-west Ethiopia	Malaria Journal	8		177	19642976
Ketema	2011	Therapeutic efficacy of chloroquine for treatment of Plasmodium vivax malaria cases in Halaba district, South Ethiopia.	Parasit Vectors	4	46	1-7	21453465
Khan	2006	Efficacy and safety of halofantrine in Pakistani children and adults with malaria caused by P. falciparum and P. vivax	SE Asian J Trop Med Public Health	37	4	613-618	17121283

References of Clinical Trials

First Author	Year	Title	Journal	Vol	Issue	Pages	Acc Number
Kinzer	2010	Active case detection, treatment of falciparum malaria with combined chloroquine and sulphadoxine/pyrimethamine and vivax malaria with chloroquine and molecular markers of antimalarial resistance in the Republic of Vanuatu	Malaria Journal	9		89	20370920
Kolaczinski	2007	Sulfadoxine-pyrimethamine plus artesunate compared with chloroquine for the treatment of vivax malaria in areas co-endemic for Plasmodium falciparum and P. vivax: a randomised non-inferiority trial in eastern Afghanistan	Trans R Soc Trop Med Hyg	101		1081-1087	17707447
Krudsood	2007	Clinical efficacy of chloroquine versus artemether-lumefantrine for Plasmodium vivax treatment in Thailand.	Korean J Parasitol	45	2	111-114	17570973
Kurcer	2004	Efficacy of chloroquine in the treatment of Plasmodium vivax malaria in Turkey.	Ann Trop Med Parasitol	98	5	447-451	15257793
Kurcer	2006	The decreasing efficacy of chloroquine in the treatment of Plasmodium vivax malaria, in Sanliurfa, south-eastern Turkey.	Ann Trop Med Parasitol	100	2	109-113	16492358
Lacy	2002	Atovaquone/proguanil therapy for Plasmodium falciparum and Plasmodium vivax malaria in Indonesians who lack clinical immunity	Clin Infect Dis	35		92-95	12384852
Lal	1982	A comparative trial of oral chloroquine and oral co-trimoxazole in vivax malaria in children	Am J Trop Med Hyg	31	3	438-440	7044160
Leang	2013	Efficacy of dihydroartemisinin-piperaquine for the treatment of uncomplicated Plasmodium falciparum and Plasmodium vivax in Cambodia, 2008-2010	Antimicrob Agents Chemother	57	9	818-826	23208711
Lee	2009	Biological resistance of hydroxychloroquine for Plasmodium vivax malaria in the Republic of Korea	Am J Trop Med Hyg	81	4	600-604	19815873
Leslie	2007	Sulfadoxine-pyrimethamine, chlorproguanil-dapsone, or chloroquine for the treatment of Plasmodium vivax malaria in Afghanistan and Pakistan: a randomized controlled trial	JAMA	297	20	2201-2209	17519409
Leslie	2008	A randomized controlled trial of 8 weeks, once weekly primaquine regimen to prevent relapse of Plasmodium vivax in Northwest Frontier Province, Pakistan	PLoS One	3	8	2861	18682739
Li	1994	Clinical trials of artemisinin and its derivatives in the treatment of malaria in China	Trans R Soc Trop Med Hyg	88		5-6	8053027
Lim	1999	Response to chloroquine of Plasmodium vivax among South Korean soldiers	Ann Trop Med Parasitol	93	6	565-8	10707102
Liu	2013	Artemisinin-naphthoquine combination versus chloroquine-primaquine to treat vivax malaria: an open-label randomized and non-inferiority trial in Yunnan Province, China	Malaria journal	12	409	doi:10.1186/	24215565
Llanos-Cuentas	2013	Tafenoquine plus chloroquine for the treatment and relapse prevention of Plasmodium vivax malaria (DETECTIVE): a multicentre, double-blind, randomised, phase 2b dose-selection study	Lancet	383	9922	1049-1058	24360369
Lon-2014	2014	Efficacy of Two versus Three-Day Regimens of Dihydroartemisinin-Piperaquine for Uncomplicated Malaria in Military Personnel in Northern Cambodia: An Open-Label Randomized Trial	PLoS One	9	3	e93138	24667662
Looareesuwan	1996	Clinical studies of atovaquone, alone or in combination with other antimalarial drugs, for treatment of acute uncomplicated malaria in Thailand	Am J Trop Med Hyg	54	1	62-66	8651372
Looareesuwan	1999a	Chloroquine sensitivity of Plasmodium vivax in Thailand.	Ann Trop Med Parasitol	93	3	225-230	10562823
Looareesuwan	1999b	Atovaquone and proguanil hydrochloride followed by primaquine for treatment of Plasmodium vivax malaria in Thailand	Trans R Soc Trop Med Hyg	93		637-640	10717754
Luxemburger	1999	Treatment of vivax malaria on the western border of Thailand.	Trans R Soc Trop Med Hyg	93		433-438	10674098

References of Clinical Trials

First Author	Year	Title	Journal	Vol	Issue	Pages	Acc Number
Machado	2003	Correlation between Plasmodium vivax variants in Belém, Pará State, Brazil and symptoms and clearance of parasitaemia.	Braz J Infect Dis	7	3	175-177	14499040
Maguire	2002	Chloroquine or sulfadoxine-pyrimethamine for the treatment of uncomplicated, Plasmodium falciparum malaria during an epidemic in Central Java, Indonesia	Ann Trop Med Parasitol	96	7	655-668	12537627
Maguire	2006	Mefloquine Is Highly Efficacious against Chloroquine-Resistant Plasmodium vivax Malaria and Plasmodium falciparum Malaria in Papua, Indonesia	Clin Infect Dis	42	8	1067-1072	16575721
Marfurt	2007	Low efficacy of amodiaquine or chloroquine plus sulfadoxine-pyrimethamine against Plasmodium falciparum and P. vivax malaria in Papua New Guinea.	Am J Trop Med Hyg	77	5	947-954	17984359
Marques	2014	Plasmodium vivax chloroquine resistance and anemia in the western Brazilian Amazon.	Antimicrob Agents Chemother	58	1	342-7	24165179
McGready	2002	The effects of quinine and chloroquine antimalarial treatments in the first trimester of pregnancy	Trans R Soc Trop Med Hyg	96	2	180-4	12055810
Mishra	2012	Monitoring antimalarial drug resistance in India via sentinel sites: outcomes and risk factors for treatment failure, 2009-2010.	Bulletin of the WHO	90		895-904	23284195
Mohapatra	2002	Atypical manifestation of Plasmodium vivax malaria	Indian Journal of Malariology	39	40940	18-25	14686106
Muhamad	2011	Monitoring of clinical efficacy and in vitro sensitivity of Plasmodium vivax to chloroquine in area along Thai Myanmar border during 2009-2010	Malaria Journal	10		44	21324161
Murphy	1993	Vivax malaria resistant to treatment and prophylaxis with chloroquine.	Lancet	341	8837		8093414
Nandy	2003	Monitoring the chloroquine sensitivity of Plasmodium vivax from Calcutta and Orissa, India.	Ann Trop Med Parasitol	97	3	215-220	12803853
Nateghpour	2007	Evaluation of Sensitivity of Plasmodium vivax to Chloroquine	Iranian Journal of Public Health	36	3	60-3	NonPubMed2
Nguyen	1993	Treatment of malaria in Vietnam with oral artemisinin	Am J Trop Med Hyg	48	3	398-402	8470777
Osorio	2007	[Assessment of the efficacy of antimalarial drugs in Tarapaca, in the Colombian Amazon basin]	Biomedica	27	1		17546230
Phan	2002	Artemisinin or chloroquine for blood stage Plasmodium vivax malaria in Vietnam.	Trop Med Int Health	7	10	858-864	12358621
Phyo	2011	Dihydroartemisinin-piperaquine versus chloroquine in the treatment of P.vivax malaria in Thailand: a randomized controlled trial	Clin Infect Dis	53		977-984	22002979
Pinto	2003	Assessment of chloroquine single dose treatment of malaria due to Plasmodium vivax in Brazilian Amazon	Revista do Instituto de Medicina Tropical	45	6	327-31	14762633
Poravuth	2011	Pyronaradine-Artesunate versus chloroquine in patients with acute P. vivax malaria: a radnomized double-blind, non-inferiority trial	PLoS One	6	1	14501	21267072
Potkar	1995	Resurgence of malaria and drug resistance in plasmodium falciparum and plasmodium vivax species in Bombay.	J Assoc Physicians India	43	5	336-338	9081964
Pukrittayakamee	2000	Therapeutic responses to different antimalarial drugs in vivax malaria.	Antimicrob Agents Chemother	44		1680-1685	10817728
Pukrittayakamee	2001	Therapeutic responses to antibacterial drugs in vivax malaria	Trans R Soc Trop Med Hyg	95		524-528	11706666
Pukrittayakamee	2010	A Comparison of Two Short-Course Primaquine Regimens for the Treatment and Radical Cure of Plasmodium vivax Malaria in Thailand	Am J Trop Med Hyg	82	4	542-547	20348496
Pukrittayakamee	1994a	Blood stage antimalarial efficacy of primaquine in Plasmodium vivax malaria	J Infect Dis	169		932-5	8133114
Pukrittayakamee	1994b	Antimalarial effects of rifampin in Plasmodium vivax malaria	Antimicrob Agents Chemother	38	3	551-514	8203846
Rajgor	2003	Efficacy of a 14 day primaquine regimen in preventing relapses in patients with Plasmodium vivax malaria in Mumbai, India	Trans R Soc Trop Med Hyg	97		438-440	15259476
Rao	1993	Efficacy and safety of halofantrine in acute malaria	J Assoc Physicians India	41	8	507-8	8294355

References of Clinical Trials

First Author	Year	Title	Journal	Vol	Issue	Pages	Acc Number
Ratcliff	2007a	Therapeutic response of multidrug-resistant Plasmodium falciparum and P. vivax to chloroquine and sulfadoxine—pyrimethamine in southern Papua, Indonesia	Trans R Soc Trop Med Hyg	101		351-359	17028048
Ratcliff	2007b	Two fixed-dose artemisinin combinations for drug-resistant falciparum and vivax malaria in Papua, Indonesia: an open-label randomised comparison	Lancet	369		757-65	17336652
Rios-2013	2013	Ten years of chloroquine efficacy for uncomplicated Plasmodium vivax malaria treatment, Turbo, Antioquia, 2002 and 2011	Biomédica	33	3	429-38	24652179
Rowland	1999	Randomized controlled trials of 5- and 14-days primaquine therapy against relapses of vivax malaria in an Afghan refugee settlement in Pakistan	Trans R Soc Trop Med Hyg	93	6	641-3	10717755
Ruebush	2003	Chloroquine-resistant Plasmodium vivax malaria in Peru.	Am J Trop Med Hyg	69	5	548-552	14695094
Saravu	2012	Plasmodium vivax remains responsive to chloroquine with primaquine treatment regimen: a prospective cohort study from tertiary care teaching hospital in southern India	Tropical Doctor	42	3	163-4	22516030
Shalini	2014	Chloroquine efficacy studies confirm drug susceptibility of Plasmodium vivax in Chennai, India	Malaria Journal	13	1	doi:10.1186/	24685286
Silachamroon	2003	Clinical trial of oral artesunate with or without high-dose primaquine for the treatment of vivax malaria in Thailand	Am J Trop Med Hyg	69	1	14-18	12932090
Singh	2000	Emergence of chloroquine-resistant vivax malaria in south Bihar (India).	Trans R Soc Trop Med Hyg	94		327	10975013
Soto	2001	Plasmodium vivax clinically resistant to chloroquine in Colombia.	Am J Trop Med Hyg	65	2	90-93	11508397
Srivastava	2008	Therapeutic responses of Plasmodium vivax and P. falciparum to chloroquine, in an area of western India where P. vivax predominates	Ann Trop Med Parasitol	102	66	71-480	18782486
Sumawinata	2003	Very high risk of therapeutic failure with chloroquine for uncomplicated Plasmodium falciparum and P. vivax malaria in Indonesian Papua.	Am J Trop Med Hyg	68	4	416-420	12875290
Sutanto	2009	Resistance to chloroquine by Plasmodium vivax at Alor in the Lesser Sundas Archipelago in eastern Indonesia	Am J Trop Med Hyg	81	2	338-342	19635895
Sutanto	2010	Evaluation of chloroquine therapy for vivax and falciparum malaria in southern Sumatra, western Indonesia	Malaria Journal	9	52		20152016
Tan	1995	Clinical response and susceptibility in vitro of Plasmodium vivax to the standard regimen of chloroquine in Thailand.	Trans R Soc Trop Med Hyg	89		426-429	7570887
Tasanor	2006	Clinical-parasitological response and in-vitro sensitivity of Plasmodium vivax to chloroquine and quinine on the western border of Thailand.	Trans R Soc Trop Med Hyg	100		410-418	16497347
Taylor	2000	Assessing drug sensitivity of Plasmodium vivax to halofantrine or choroquine in southern, central Vietnam using an extended 28-day in vivo test and polymerase chain reaction genotyping.	Am J Trop Med Hyg	62	6	693-697	11304056
Taylor	2001	Chloroquine/doxycycline combination versus chloroquine alone, and doxycycline alone for the treatment of Plasmodium falciparum and Plasmodium vivax malaria in northeastern Irian Jaya, Indonesia.	Am J Trop Med Hyg	64	5	223-228	11463107
Teka	2008	Chloroquine-resistant Plasmodium vivax malaria in Debre Zeit, Ethiopi	Malaria journal	7	220	1-8	18959774
Than	1995	Development of resistance to chloroquine by Plasmodium vivax in Myanmar.	Trans R Soc Trop Med Hyg	89		307*308	7660445
Tjitra	2002	Therapeutic efficacies of artesunate-sulfadoxine-pyrimethamine and chloroquine-sulfadoxine-pyrimethamine in vivax malaria pilot studies: relationship to Plasmodium vivax dhfr mutations	Antimicrob Agents Chemother	46	12	347-3953	12435700

References of Clinical Trials

First Author	Year	Title	Journal	Vol	Issue	Pages	Acc Number
Tjitra	2012	Efficacy and safety of artemisinin-naphthoquine versus dihydroartemisinin-piperaquine in adult patients with uncomplicated malaria: a multi-centre study in Indonesia	Malaria Journal	1		e153	22554203
Trukillo	2002	[Adequate clinical and parasitological Plasmodium vivax response to chloroquine in Colombia (Turbo, Antioquia), 2001]	Infectio	6	1	21-26	NonPubMed1
Tulu	1996	Failure of chloroquine treatment for malaria in the highlands of Ethiopia.	Trans R Soc Trop Med Hyg	90		556-557	8944273
Valecha	2006	Therapeutic efficacy of chloroquine in Plasmodium vivax from areas with different epidemiological patterns in India and their Pvdhfr gene mutation pattern.	Trans R Soc Trop Med Hyg	100		831-837	16513151
Valibayov	2003	Clinical efficacy of chloroquine followed by primaquine for Plasmodium vivax treatment in Azerbaijan.	Acta Trop	88		99-102	12943984
Vijaykadga	2004	Assessment of therapeutic efficacy of chloroquine for vivax malaria in Thailand.	SE Asian J Trop Med Public Health	35	3	566-569	15689067
Villalobos	2000	In-vivo sensitivity of Plasmodium vivax isolates from Rond nia (western Amazon region, Brazil) to regimens including chloroquine and primaquine.'	Ann Trop Med Parasitol	94	8	749-758	11214093
Walsh	1999	Randomized dose-ranging study of the safety and efficacy of WR 238605 (Tafenoquine) in the prevention of relapse of Plasmodium vivax malaria in Thailand	The Journal of Infectious Diseases	180	4	1282-7	10479159
Walsh	2004	Randomized trial of 3-dose regimens of tafenoquine (WR238605) versus low-dose primaquine for preventing Plasmodium vivax malaria relapse	Clin Infect Dis	39		1095-1103	15486831
Wilairatana	1999	Efficacy of primaquine regimens for primaquine-resistant Plasmodium vivax malaria in Thailand	Am J of Trop Med Hyg	61	6	973-7	10674681
